# Setting of the magnetic structure of chiral kagome antiferromagnets by a seeded spin-orbit torque

**DOI:** 10.1126/sciadv.abo5930

**Published:** 2022-06-15

**Authors:** Banabir Pal, Binoy K. Hazra, Börge Göbel, Jae-Chun Jeon, Avanindra K. Pandeya, Anirban Chakraborty, Oliver Busch, Abhay K. Srivastava, Hakan Deniz, James M. Taylor, Holger Meyerheim, Ingrid Mertig, See-Hun Yang, Stuart S. P. Parkin

**Affiliations:** 1 Max Planck Institute of Microstructure Physics, Weinberg 2, 06120 Halle, Germany.; 2 Institute of Physics, Martin Luther University Halle-Wittenberg, 06099 Halle, Germany.

## Abstract

The current-induced spin-orbit torque switching of ferromagnets has had huge impact in spintronics. However, short spin-diffusion lengths limit the thickness of switchable ferromagnetic layers, thereby limiting their thermal stability. Here, we report a previously unobserved seeded spin-orbit torque (SSOT) by which current can set the magnetic states of even thick layers of the chiral kagome antiferromagnet Mn_3_Sn. The mechanism involves setting the orientation of the antiferromagnetic domains in a thin region at the interface with spin currents arising from an adjacent heavy metal while also heating the layer above its magnetic ordering temperature. This interface region seeds the resulting spin texture of the entire layer as it cools down and, thereby, overcomes the thickness limitation of conventional spin-orbit torques. SSOT switching in Mn_3_Sn can be extended beyond chiral antiferromagnets to diverse magnetic systems and provides a path toward the development of highly efficient, high-speed, and thermally stable spintronic devices.

## Teaser

The magnetic state of very thick chiral antiferromagnetic films can be set by a seeded spin-orbit torque.

## INTRODUCTION

The discovery of current-induced torques for magnetic switching has major implications for the development of novel spintronic devices (*1*, *2*). In particular, spin-orbit torques (SOTs) (*2*–*7*) derived from spin currents have been shown to be effective in switching magnetization. Typically, however, only thin magnetic layers can be switched because of short spin diffusion lengths (*8*). Therefore, thermally stable nanospintronic devices are difficult to create because the volume of the magnetic material and, consequently, the magnetic anisotropy energy is limited. Current-induced torques that could switch thick magnetic layers could overcome this dilemma. Even more interesting would be the current-induced switching of antiferromagnets (AFs), whether collinear or noncollinear, because AFs lack long-range dipole fields that otherwise cause interactions within and between devices (*9*–*11*).

The field of AF spintronics has grown substantially over the past decade with numerous demonstrations of current-induced switching involving predominantly collinear AFs (*10*–*22*). Recently, however, there has been considerable interest in noncollinear chiral AFs (*23*–*25*). These systems display a nonvanishing Berry phase that was theoretically predicted (*26*) to give rise to several unusual properties including anomalous Hall effect and magneto-optical Kerr signal even with a nearly fully compensated magnetic structure. These properties were subsequently observed in the chiral kagome AFs Mn_3_*X* (*X* = Sn, Ge) (*24*, *25*, *27*, *28*). Recently, it was shown that current passing through a heavy metal interfaced with Mn_3_Sn can modify its magnetic state (*29*, *30*). The mechanism was attributed to a conventional SOT derived from spin currents generated via a spin Hall effect (SHE) in the heavy metal layer (*29*, *30*). This result was unexpected because the layers of Mn_3_Sn were much thicker than the spin diffusion length (*31*). Here, we show that the switching mechanism of Mn_3_Sn goes beyond a simple SOT mechanism and that thermal effects play a key role. The mechanism involves the setting of the AF domain configuration at the interface of Mn_3_Sn with a heavy metal layer in which a spin-current “seeds” the subsequent setting of the domain configuration of the entire AF. The process also involves bringing the temperature of the AF above its ordering temperature and then cooling it in the presence of the SOT provided by the spin current. In this regard, this mechanism is analogous to that of the formation of exchange bias fields at the interface between an AF and a ferromagnet (FM). We substantiate our hypothesis by experiments in which we show that current pulses whose fall time is too short do not allow for switching of the entire AF layer. Furthermore, we demonstrate the first evidence of high-speed magnetization reversal of a chiral AF using a nanosecond current pulse in conjunction with a bias current. Our results of nanosecond magnetization switching mediated by a novel seeded SOT (SSOT) mechanism provide a new understanding of the switching process of chiral AFs and pave the way for the realization of thermally stable AF spintronic devices with low power and high speed.

## RESULTS AND DISCUSSION

### Magnetic ground states of Mn_3_Sn and electrical switching using protocol I

**Fig. 1.**
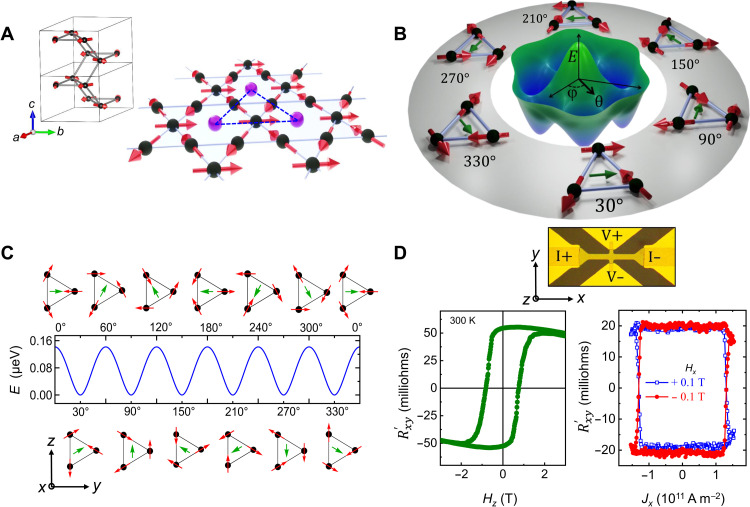
Sixfold magnetic ground-state and current-induced switching of Mn_3_Sn. (**A**) Mn atoms (black spheres) in Mn_3_Sn constitute a kagome lattice plane where Sn atoms (purple spheres) occupy the center of each hexagon. The blue dashed lines, connecting Sn atoms, indicates the easy axes. (**B**) Three-dimensional (3D) energy landscape of the sixfold degenerate magnetic configuration of Mn_3_Sn where φ and θ denote the rotation of the net moment ***m*** (green arrow) within and out of the kagome plane, respectively. (**C**) Six energetically degenerate ground states and intermediate excited states of a representative Mn triangle along with the 2D energy landscape as φ is varied (for θ = 0). (**D**) Left: Anomalous Hall resistance of a 40-nm Mn_3_Sn/8-nm W/3-nm TaN sample measured at 300 K with 1-mA read current. Right: Current-induced switching of the same device measured at 300 K using protocol I for positive and negative *H_x_* = 0.1 T. Top: Optical micrograph of Hall bar device. The magnetic fields *H_z_* and *H_x_* are applied along “*z*” and “*x*,” respectively, and the current is along *x*.

Mn_3_Sn crystallizes in a hexagonal lattice with a kagome AF structure, as illustrated in Fig. 1A. A vector Dzyaloshinskii-Moriya interaction, together with a Heisenberg exchange interaction, results in a coplanar 120° AF ordering (*26*, *32*). The presence of single-ion magnetic anisotropy breaks this high-symmetry spin order and produces three in-plane magnetic easy axes (*32*) (the dashed lines in Fig. 1A). This results in small deviations of the orientations of the Mn moments and, consequently, a small in-plane magnetic moment ***m*** (0.002 μ_B_/f.u.) (*33*). Minimizing the energy, corresponding to the Hamiltonian

$$H=A\sum_{<i,j>}m_{i}∙m_{j}+D\sum_{<i,j>}d_{\mathrm{ij}}∙(m_{i}\times m_{j})-K\sum_{i}{\left(k_{i}∙m_{i}\right)}^{2}$$

with parameters taken from previous work (*29*), shows that there are six energetically degenerate magnetic ground states (Fig. 1, B and C) characterized by ***m*** pointing along the polar angles φ = 30°, 90°, …,330° (Fig. 1C). The corresponding local energy maxima are the unstable equilibrium configurations with φ = 60°,120°, …,360° (Fig. 1C). These magnetic configurations, as well as the energy barriers, will play a key role in the current-induced SSOT switching mechanism.

Highly textured thin films of Mn_3_Sn, ranging from 30 to 100 nm thick, with and without heavy metal overlayers of W, were grown on top of an Al_2_O_3_ ($1\bar{1}02$) substrate using magnetron cosputtering (see Materials and Methods). For this growth orientation, the kagome planes are oriented perpendicular to the substrate such that an anomalous Hall effect (AHE) can be observed (*24*, *25*). For a 40-nm-thick film, the anomalous Hall resistance, $R_{\mathrm{xy}}^{\prime }$, switches between ±50 milliohms at 300 K (Fig. 1D, left) with a coercivity ~1 T. Referencing Fig. 1C, this corresponds to a field-induced switching of ***m*** between the φ = 90° and 270° states. Note that $R_{\mathrm{xy}}^{\prime }$ is the measured value of the anomalous Hall resistance, *R_xy_*, minus a constant background signal that has been subtracted.

Current-induced switching experiments are carried out using long (~100 ms) current pulses, together with a magnetic field, *H_x_*, applied along the current direction (*x* direction in Fig. 1D, top). The resulting AF state is probed by measuring *R_xy_* approximately 0.5 s after the end of the writing pulse with a 1-mA read current (protocol I). We observe clear evidence for hysteretic current-induced switching at *H_x_* = ± 0.1 T (Fig. 1D, right) where $R_{\mathrm{xy}}^{\prime }$ switches between ±20 milliohms, approximately 60% smaller than that found for field-induced switching (Fig. 1D, left). These results are similar to those reported previously for such long current pulses (*29*, *30*). We define the current-induced switching ratio ξ as the ratio of the change in $R_{\mathrm{xy}}^{\prime }$ engendered by current versus that found from magnetic field–induced switching $\left(\xi =\frac{R_{\mathrm{xy}}^{\prime }(\text{current})}{R_{\mathrm{xy}}^{\prime }\;(\text{field})}\right)$. Using the Landau-Lifshitz-Gilbert equation including a SOT term and atomistic simulations (see Materials and Methods), we could reproduce the experimental observation of current-induced switching (fig. S8). However, the calculated switching current density (*J*_c_), is found to be much larger than that measured experimentally, as was also found in a previous report (*30*). Thus, a new mechanism is called for.

### Electrical switching using protocol II and the role of joule heating

**Fig. 2.**
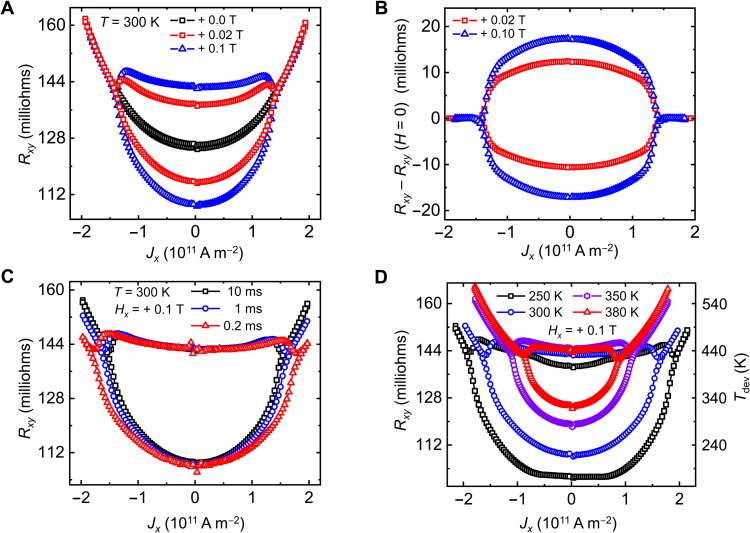
Current-induced switching of Mn_3_Sn using protocol II. (**A**) Current-induced switching at 300 K using protocol II for different in-plane magnetic fields applied parallel to the current direction (*H_x_*). (**B**) Background estimated from zero-field switching experiments was subtracted to plot the dependence of *R_xy_* on *J*_c_. (**C**) Switching experiments at 300 K for three different pulse lengths,τ*_L_*. (**D**) Similar switching experiments at different set temperatures for a selected pulse length,τ*_L_* ~ 1 ms. The device temperature during the switching process can be quantitatively estimated from the variation of the *R_xy_* component, as shown in the right axis of the Fig. 2D. These results show that switching always takes place at the same temperature.

To explore the current-induced switching mechanism, both the writing of the device and its readout were simultaneously performed using a single current pulse (protocol II) so that the transient state of the system during the writing process is probed rather than the state after thermal relaxation as in protocol I (see the Supplementary Materials). The *R_xy_* versus *J_x_* curves for different *H_x_* using this new protocol II (Fig. 2A) are distinguishable from their protocol I counterpart (Fig. 1D, right). For example, sharp transitions at *J_x_* = ± *J*_c_ that were seen in protocol I vanish in protocol II. Each *R_xy_* versus *J_x_* curve in protocol II (Fig. 2A) exhibits a quadratic background arising from the intermixing of a *R_xx_* component with *R_xy_*. As is often found in Hall measurements, a slight misalignment of the Hall contacts leads to a finite *R_xx_* component within *R_xy_*. It is observed that *R_xx_* increases rapidly with applied current that we surmise is due to an increase in the device temperature by joule heating. *R_xx_* enables us to thereby measure the sample temperature directly during the application of the current pulse. The *R_xx_* component was determined by measuring *R_xy_* in 0 T (Fig. 2A), because there is no change in the *R_xy_* contribution in zero field. After subtracting the *R_xx_* component, the dependence of *R_xy_* on *J_x_* is shown in Fig. 2A.

To further investigate the role of joule heating, we carried out additional experiments using protocol II at different set temperatures. Switching experiments at room temperature for three different current pulse lengths (Fig. 2C) show a lower *J*_c_ for longer pulses. As the set temperature is increased, we find that *J*_c_ is systematically decreased for the same current pulse length (Fig. 2D). In all these experiments, we find that the switching always takes place at exactly the same value of *R_xy_* (~144 milliohms) independent of the current pulse length, set temperature, and magnetic field (Fig. 2, C, D, and A, respectively). As discussed in the previous section, the total *R_xy_* has an extrinsic *R_xx_* contribution together with an intrinsic *R_xy_*. At *J_x_* = ± *J*_c_, the intrinsic *R_xy_* becomes zero (Fig. 2B), and the total *R_xy_* is completely dominated by the *R_xx_* contribution. Hence, the measured value of *R_xy_* quantitatively serves as a measure of the device temperature. Therefore, the switching at a particular value of *R_xy_* corresponds to a fixed critical temperature (T*). Our estimate of T* from the change in *R_xy_* (see inset of fig. S9A) shows that T* is approximately 435 K, which is close to the Néel temperature of Mn_3_Sn (*25*, *33*–*36*). The universality of switching at T* = 435 K indicates that heating of the Mn_3_Sn layer close to the Néel temperature plays a key role in the switching process. On the other hand, we note that a previous work (*30*) reports much lower Néel temperatures in their ultrathin Mn_3_Sn films, which possibly explains why less heating of their films is needed for current-induced switching that they suggest arises from a pure SOT mechanism.

### Inconsistencies with pure SOT switching

**Fig. 3.**
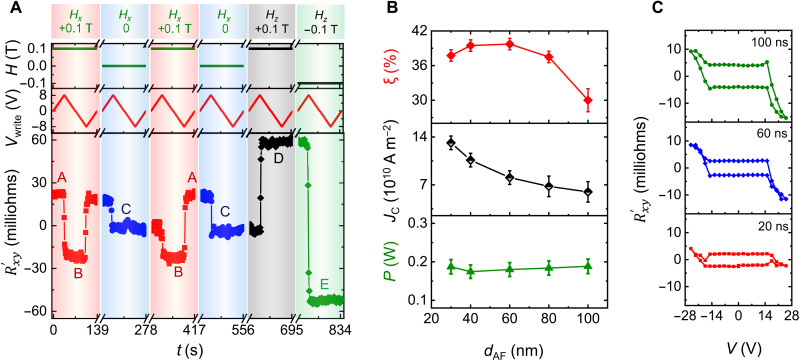
Inconsistencies with pure SOT switching. (**A**) Switching experiments using protocol I for in-plane magnetic field parallel to the current direction (*H_x_*) and an out-of-plane magnetic field (*H_z_*). (**B**) Variation of the switching ratio, ξ, the critical current density in the W layer for switching, *J*_c_, and the critical power in the device, *P*_c_, as a function of, *d*_AF_, the Mn_3_Sn layer thickness. (**C**) $R_{\mathrm{xy}}^{\prime }$ versus applied voltage across the device for three different current pulse lengths. ξ is very small in these cases.

Additional switching experiments using protocol I are carried out to demonstrate that a pure SOT mechanism cannot account for all the observed features of our switching experiments. Figure 3A shows switching of Mn_3_Sn in the presence of different fields. First at *H_x_* = 0.1 T, the device switches between two distinct states labeled by “A” and “B” (red shaded region). Thereafter, when the same experiments are carried out in the absence of any field, the device switches to a state “C” with a zero $R_{\mathrm{xy}}^{\prime }$ for a current exceeding ±*J*_c_. Therefore, state C corresponds to a demagnetized state resulting from heating of the device beyond its ordering temperature, as discussed previously. It has been proposed recently that such a change might arise from a “chiral spin rotation” (*30*) enabled by SOT. We have carried out switching experiments in Mn_3_Sn without any W layer (spin current source) to rule out such a possibility (see fig. S11B). Our results show that a randomization of the magnetic domains can take place even without a W layer and arises once the temperature of the device reaches the ordering temperature (T*).

Furthermore, in the presence of a small out-of-plane magnetic field, *H_z_* = ±0.1 T, that is 10 times smaller than the coercive field of the device (see Fig. 1D, left), we observe current-induced switching between two states (labeled as “D” and “E”) at similar critical currents (±*J*_c_) to that found above for switching using an in-plane field. However, the magnitude of the change in $R_{\mathrm{xy}}^{\prime }$ is much larger and is comparable to that found for pure field-induced switching (at a much larger field). The result, that all switching takes place with the same *J*_c_, highlights that switching takes place when the sample temperature reaches close to the AF ordering temperature. This supports our hypothesis that heating of the sample is a necessary prerequisite for the switching mechanism.

More experiments using protocol I were carried out for devices with varying Mn_3_Sn thicknesses, *d*_AF_ (from 30 to 100 nm) with the same W thickness (8 nm) at *H_x_* = + 0.1 T. The corresponding *J*_c_, critical switching power (*P*_c_), and ξ are plotted in Fig. 3B. We find that *J*_c_ decreases with increasing *d*_AF_, whereas ξ is nearly independent of *d*_AF_ (Fig. 3B). This is inconsistent with a pure SOT mechanism, which would rather require an increasing spin current density magnitude with increasing *d*_AF_ (*37*). On the other hand, the critical switching power (*P*_c_) is independent of thickness consistent with a heating-induced switching mechanism when the needed power is limited by surface and interface cooling. Last, we note that the spin diffusion length in Mn_3_Sn has been reported to be very small [~1 nm (*31*)], making it unlikely that a pure SOT can switch samples whose thickness are two orders of magnitude higher than the nominal spin diffusion length.

Further evidence for the role of temperature comes from experiments in which much shorter, nanosecond-long, current pulses are used. Considering that the time scale associated with conventional SOT switching of FMs is ~10 ns (*38*), the pulses that are longer than 10 ns should give rise to an ξ that is independent of pulse length. On the contrary, we observe that ξ is substantially reduced for 100-ns and shorter pulses (Fig. 3C) compared to the 100-ms pulses (Fig. 1D, right). Once again, conventional SOT cannot account for these results.

### SSOT mechanism

**Fig. 4.**
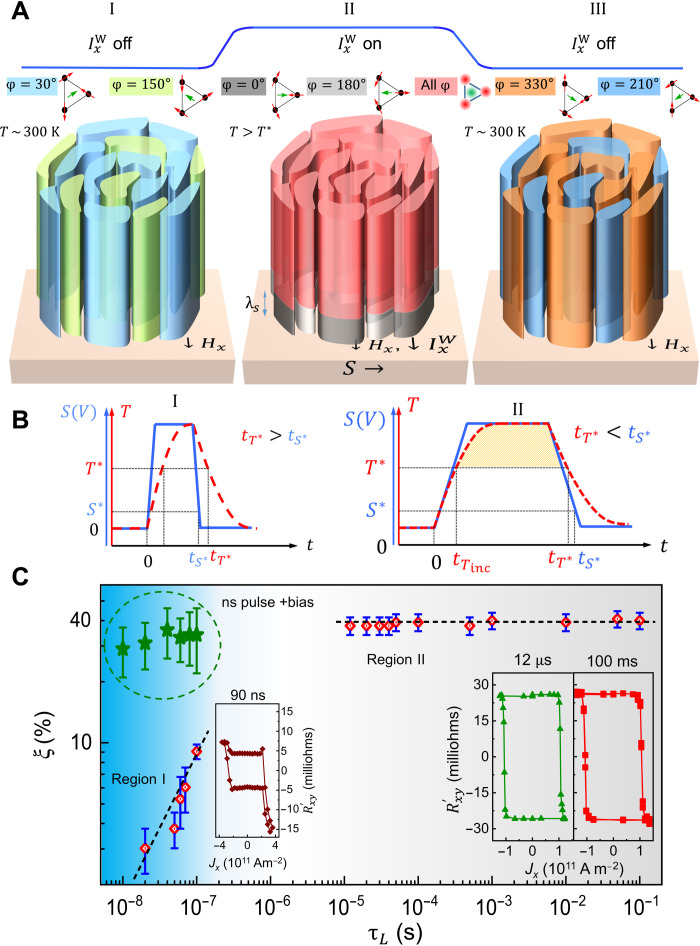
SSOT mechanism. (**A**) Schematic illustration of SSOT. Shape of the domains is for pictorial purposes. Initial spin configurations with φ = 30°/150° are shown in (I). Excited spin configurations are shown in (II) where all magnetic domains thermally fluctuate (red region). Near interface domains are oriented along φ = 0°/180° (gray) due to SOT. Final spin configurations are shown in (III) with φ = 330°/210°. (**B**) Schematics of the switching with current pulses of (i) narrower τ*_L_* with fast fall time (left) and (ii) longer τ*_L_* with slow fall time (right). T* represents the ordering temperature, and S* denotes the critical spin current density necessary to switch when the system is above T*. Time required for *T* and *S* to relax below T* and S* are given by *t*_T*_ and *t*_S*_, respectively. (**C**) Dependence of ξ on τ*_L_* and distinct fall times. For longer τ*_L_* with slow fall times (region II), Mn_3_Sn cools below T* in the presence of S* because *t*_S*_ > *t*_T*_ and hence, ξ ~ 40%. For narrower τ*_L_* with fast fall times (region I), Mn_3_Sn cools below T* in the absence of S* because *t*_S*_ < *t*_T*_ and hence, ξ ≪ 40%. Switching with nanosecond pulses and bias current leads to ξ ~ 40%.

Our hypothesis that can account for the experimental data presented above is illustrated schematically in Fig. 4 (A and B). When a writing pulse is applied, the SOT derived from the spin current is too weak to switch the entire volume of the Mn_3_Sn layer, and the writing pulse rather increases the device temperature. When the device temperature exceeds the AF ordering temperature, T*, all the AF domains in Mn_3_Sn are randomized. Once the system cools below T* in the presence of a critical spin current density (S*), a thin region (thickness~λ*_s_*) at the interface with W (gray region near the interface in case II of Fig. 4A) will be switched toward either φ = 0° or 180° depending on the sign of the spin current and the direction of *H_x_*. This thin region constitutes the “seed” layer. The interface region subsequently seeds the domain configuration of the bulk of the AF layer (red region in case II) as the entire layer cools. We note that the SSOT mechanism is analogous to the formation of an exchange bias field when an AF layer is coupled to a FM layer. When such a bilayer is cooled from above the AF blocking temperature, the exchange interaction provided by the magnetized FM layer across the atomically thin AF/FM interface sets the AF domain configuration at the interface, seeding the entire AF layer as it is cooled (*23*, *39*, *40*). In our case, the SOT provided by a spin current plays a role analogous to the FM in the case of exchange bias. We also note that heat plus magnetic field has been used, on the one hand, to nucleate non collinear spin textures, namely, skyrmions (*41*–*43*), and, on the other hand, more conventionally to switch magnetic bits for high-density magnetic recording (*44*).

### Importance of fall time in SSOT mechanism

The SSOT mechanism is only effective if the device cools through T* in the presence of an SOT arising from a critical spin current (S*). Thus, the fall time of the current pulse plays an important role. If the writing current switches off abruptly, then the spin current goes to zero while the device is still above T*. This leads to a lower magnitude in switching (case *t*_T*_ > *t*_S*_ in Fig. 4B, left). On the contrary, if the current pulse fall time is sufficiently long, then the Mn_3_Sn layers cools while the interface layer configuration is set by the spin current. This leads to a higher magnitude of switching (case *t*_T*_ < *t*_S*_ in Fig. 4B, right).

Variable pulse length–dependent switching experiments are carried out to differentiate these two distinct scenarios. Two types of write current pulses are used, in which, first, a pulse length is varied from 20 to 100 ns with a short fall time of 750 ps (region I in Fig. 4C), and second, a pulse length is varied from 12 μs to 100 ms with a longer fall time of ~20 ns (region II in Fig. 4C). In the latter case, we find that the system always exhibits the same switching ratio ξ ~ 40%, irrespective of the pulse length. By contrast, in the first case, ξ ≪ 40%. This small ξ is due to either incomplete heating and/or *t*_T*_ > *t*_S*_ (Fig. 4B, left). We find from finite element modeling that the device reaches T* in less than ~100 ns with the applied current densities used in our experiments (see fig. S16), therefore ruling out the possibility of incomplete heating. However, if the current is turned off abruptly, then the time for the sample to cool noticeably is ~10 to 20 ns. Thus, in region I (ξ ≪ 40%), we are always in the *t*_T*_ > *t*_S*_ regime. However, in the case of region II, the fall time is longer and hence we are in the *t*_T*_ < *t*_S*_ regime, which leads to a ratio ξ ~ 40%. Thus, consistent with our hypothesis, when the fall time of the current pulse is too short, there is incomplete switching because the spin current that is needed to seed the AF domain configuration of the entire layer has been switched off while the device temperature was too high.

### High-speed switching with nanosecond current pulses and a bias current

Our experiments have shown that although the time scale associated with AF magnetization dynamics is typically much faster than FMs, we observe only partial magnetization reversal in Mn_3_Sn when the fall time of the writing current pulse is shorter than the cooling time (*t*_T*_ > *t*_S*_). To overcome this limitation, we carry out switching experiments with short current pulses (10 to 100 ns) in combination with a bias current (see fig. S20) that provides the required spin-current density (S*). We then find that current pulses as short as 10 ns switch Mn_3_Sn with a switching efficiency (ξ) as large as that for a very long (100 ms) writing pulse (Fig. 4C, top of region I).

We note that the magnetization reversal of chiral AFs using short nanosecond current pulses has not previously been realized and that the short time scales that we have achieved for the current-induced switching of AF Mn_3_Sn are comparable to those reported for conventional spin-transfer torque (STT)-induced magnetization reversal of FMs (*45*, *46*). We also note that there are several strategies to minimize the power consumption used in the SSOT switching, including, especially, the exploration of different materials with regard to optimizing the ordering temperature of the material while, at the same time, maintaining thermal stability.

### Role of in-plane magnetic field in SSOT switching

**Fig. 5.**
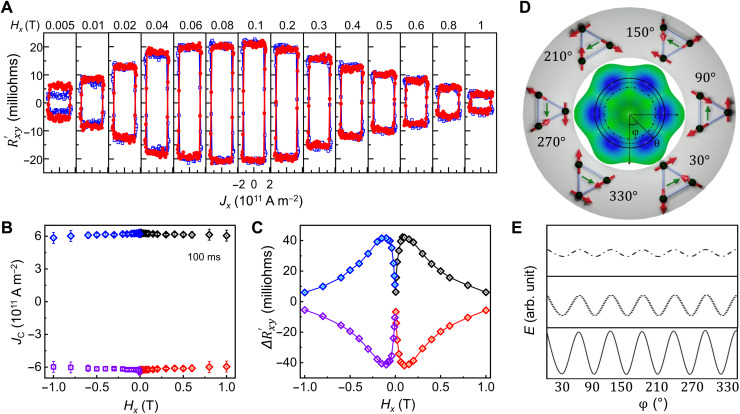
Current-induced switching in the presence of different in-plane bias fields *H_x_*. (**A**) Variation of $R_{\mathrm{xy}}^{\prime }$ on *H_x_* during current-induced switching. (**B** and **C**) Dependence of *J*_c_ (B) and ${\Delta R}_{\mathrm{xy}}^{\prime }$ (C) on *H_x_*. Black (+*H_x_*, −*J_x_*), red (+*H_x_*, *+J_x_*), purple (−*H_x_*, *−J_x_*), and blue (−*H_x_*, *+J_x_*) show negative and positive current sweep for positive and negative *H_x,_* respectively. For details, check fig. S17. (**D** and **E**) Magnetic energy landscape of Mn_3_Sn. When **m** lies within the kagome plane (θ = 0), the total energy (*E*) exhibits a sixfold degeneracy, as a function of φ, and the energy barrier between the ground state and the excited state is high (solid line). However, if **m** tilts out of the kagome plane (θ ≠ 0), then the energy barrier between the ground and excited states decreases progressively with increase in θ [bottom to top in (E); from solid line to dotted line to dashed-dotted line in (D) and (E)]. This favors a thermal randomization of the AF domain population and will lead to a smaller ${\Delta R}_{\mathrm{xy}}^{\prime }$ during switching.

Because the SSOT-mediated electrical switching in Mn_3_Sn is distinct from conventional SOT, we expect that the role of in-plane magnetic fields will also be distinct. In Fig. 5A, the in-plane field (*H_x_*) dependence of $R_{\mathrm{xy}}^{\prime }$ is displayed and the corresponding *J*_c_ and change in Hall resistance ($\text{∆}R_{\mathrm{xy}}^{\prime }$) for different *H_x_* is shown in Fig. 5 (B and C, respectively; see fig. S17 for $\text{∆}R_{\mathrm{xy}}^{\prime }$ and *J*_c_ estimation). We find that *J*_c_ remains constant and has no dependence on *H_x_* (Fig. 5B). On the other hand, $\text{∆}R_{\mathrm{xy}}^{\prime }$ exhibits an intriguing nonmonotonic dependence on *H_x_* (Fig. 5C). For fields close to zero, $\text{∆}R_{\mathrm{xy}}^{\prime }$ is negligibly small. However, with increasing *H_x_*, $\text{∆}R_{\mathrm{xy}}^{\prime }$ increases until it reaches a maximum value near $H_{x}^{\text{max}}$ (~0.1 T) beyond which it decreases. These dependences of *J*_c_ and $\text{∆}R_{\mathrm{xy}}^{\prime }$ on *H_x_* are distinct from conventional SOT switching of an FM (see fig. S18) where *J*_c_ rather decreases monotonically with increasing *H_x_* (because the damping-like torque is supplemented by *H_x_*) and $\text{∆}R_{\mathrm{xy}}^{\prime }$ remains constant, independent of *H_x_*. From atomistic calculations, we show that the initial increase in ${\Delta R}_{\mathrm{xy}}^{\prime }$ with *H_x_* is caused by a slight tilting of the uncompensated moment ***m*** out of the kagome plane. This induces a torque that increases the larger is *H_x_* (see fig. S8 for details). The decrease of ${\Delta R}_{\mathrm{xy}}^{\prime }$ beyond *H_x_* = 0.1 T can be qualitatively explained as follows: As *H_x_* is increased (>0.1 T), the out-of-kagome plane tilts of ***m*** become large. Therefore, the energy barrier between the different ground states in the energy landscape (versus φ) diminishes (see Fig. 5, D and E, for a schematic illustration), thereby favoring thermally assisted randomization of other domain configurations. This effectively decreases $\text{∆}R_{\mathrm{xy}}^{\prime }$.

### Summary

In conclusion, we have shown that current-induced switching of thick layers of the chiral kagome AF, Mn_3_Sn, cannot be accounted for by conventional SOT. These layers must be heated close to their ordering temperature and must cool down in the presence of a spin current that seeds the magnetic configuration of the entire layer. This SSOT mechanism can also be applied to a wide variety of ferro- and ferrimagnetic systems and can overcome the bottleneck to many spintronic technologies in which thick magnetic layers are needed for sufficient thermal stability. Our finding of nanosecond magnetization reversal paves the way for realizing AF spintronic devices that use low power but operate at high speed.

## MATERIALS AND METHODS

### Thin-film preparation

Thin films of Mn_3_Sn with and without heavy metal [Al_2_O_3_/Mn_3_Sn (*t* = thickness)/W (8 nm)/TaN (3 nm)] were deposited on Al_2_O_3_ $\left(1\bar{1}02\right)$ substrates by means of the d.c. magnetron sputtering technique using a homemade ultrahigh vacuum magnetron sputtering system with a chamber base pressure of ~1 × 10^−9^ torr. Mn_3_Sn layers with five different thicknesses were used in this study (*t* = 30, 40, 60, 80, and 100 nm). Atomically flat Al_2_O_3_ substrates were first prepared by a wet etching procedure followed by a heat treatment at 1200°C for 4 hours. Then, Mn_3_Sn thin films were cosputtered from Mn and Sn targets at 200°C followed by in situ annealing at 350°C for 15 min. Further, an 8-nm-thick W layer was deposited on top of the Mn_3_Sn layer at room temperature. Last, all films were capped with a 3-nm TaN layer to prevent oxidation. A Ta (5 nm)/CoFeB (1 nm)/MgO (2 nm)/Ta (3 nm) film stack was also sputtered on a SiO_2_ (25 nm)/Si (001) substrate and annealed ex situ (300°C for 30 min).

### Thin-film characterization

Rutherford backscattering techniques were used to determine the composition of each film. The stoichiometry of Mn_3_Sn thin films that were later used for switching and other experiments was estimated to be close to Mn_3_Sn_0.94 ± 0.05_. This slight deficiency in Sn is known to help stabilizing the hexagonal phases of Mn_3_Sn (*47*). A detailed crystallographic analysis was carried out to understand the crystalline orientations of the thin films using a Bruker d8 discover diffractometer. Atomic force microscopy measurements were performed to probe the surface topography. The magnetic properties of the prepared films were characterized by a MPMS3 SQUID magnetometer. The temperature-dependent measurements of the longitudinal resistivity and the anomalous Hall resistivity were carried out in a physical property measurement system (PPMS).

### Device fabrication

The prepared films were patterned into Hall bar geometries with different lengths (25, 50, and 100 μm) and widths (5, 10, and 10 μm) using conventional photolithography techniques (365-nm maskless laser writer; MLA150, Heidelberg). The patterned devices were then etched by Ar ion beam milling. The etching profiles were in situ analyzed by secondary ion mass spectroscopy to control the etch-stop point. For the electrical characterization of the devices, Ti (2 nm)/Au (100 nm) was used for the contacts (conventional photolithography and lift-off process were used). A microscopic image of the fabricated Hall bar that was used for the measurements is shown in the top of Fig. 1D.

### Electrical switching measurement

The electrical switching experiments were performed in a PPMS and probe station system. Two distinct switching protocols were used in these investigations. In protocol I, first, a write pulse of variable pulse length was used. After a specific delay period (0.5 s), the resultant magnetic orientations were probed by measuring the transverse voltage in the Hall bar with a 1-mA d.c. read current. In switching protocol II, a single current pulse is used for both writing and simultaneous reading.

### Atomistic simulations of the magnetic texture

The magnetic Hamiltonian is

$$H=A\sum_{<i,j>}m_{i}∙m_{j}+D\sum_{<i,j>}d_{\mathrm{ij}}∙(m_{i}\times m_{j})-K\sum_{i}{\left(k_{i}∙m_{i}\right)}^{2}-\mu_{0}M_{s}\sum_{i}(m_{i}∙B)$$

where *A* = 23 meV characterizes the exchange interaction, *D* = 1.6 meV the Dzyaloshinskii-Moriya interaction (DMI) and *K* = 0.17 meV the anisotropy. The ***m****_i_* are unit vectors corresponding to the orientation of the magnetic moment with magnitude *M*_s_ = 3μ*_B_* at site *i. ****B*** is the magnetic field. To simulate the switching behavior of the interfacial layer, we consider a single kagome layer with three Mn atoms in the unit cell. We propagate the Landau-Lifshitz-Gilbert equation, including a SOT term for every magnetic moment (*48*)

$$\overset{\cdot }{m}=-\gamma \;m\times B_{\text{eff}}+\alpha \;m\times \overset{\cdot }{m}+\frac{\hbar \gamma \theta_{\text{SH}}}{2em_{s}d}j\;m\times (s\times m)$$

Here, γ is the gyromagnetic ratio of an electron. The first term characterizes a precession of the magnetic moment around its effective magnetic field $B_{\text{eff}}=-\frac{1}{M_{s}}\frac{\delta H}{\delta m}$. The second term accounts for the damping and is quantified by the Gilbert damping α = 0.003. The third term is the SOT. It is generated by a charge current density *j* that we apply along *x* so that the SHE generates a spin current *j*θ_SH_ that is injected into the magnet with spins oriented along ***s = y***. The efficiency is characterized by the spin Hall angle θ_SH_ = 0.1, the magnetization density *m_s_* = 6*M*_s_/*V*, and the thickness *d* = 40 nm. All parameters have been chosen as in (*29*).

## Supplementary Materials

### This PDF file includes:

Supplementary Text Figs. S1 to S20 References
