# Supplementary material for: Setting of the magnetic structure of chiral kagome antiferromagnets by a seeded spin-orbit torque
Source: Sci Adv. 2022 Jun 15;8(24):eabo5930. doi: 10.1126/sciadv.abo5930 (PMC9200275; doi:10.1126/sciadv.abo5930)
Supplement: Supplementary file 1 — Supplementary Text Figs. S1 to S20 References [file sciadv.abo5930_sm.pdf]

Science Advances
AAAS

Supplementary Materials for

Setting of the magnetic structure of chiral kagome antiferromagnets by a seeded spin-orbit torque

Banabir Pal *et al.*

Corresponding author: Stuart S. P. Parkin, stuart.parkin@mpi-halle.mpg.de

Sci. Adv. **8**, eabo5930 (2022)
DOI: 10.1126/sciadv.abo5930

This PDF file includes:

Supplementary Text
Figs. S1 to S20
References

Electrical switching measurement

The electrical switching experiments were performed in a PPMS (Quantum Design, Inc.) and probe station (Lakeshore cryotronics) system. Two distinct switching protocols were used in these investigations. In protocol I (Fig. S1 A), first a write pulse of variable pulse length [switching scheme 1, 2 and 3 in Fig. S2] was used. After a specific delay period (0.5 s), the resultant magnetic orientations were probed by measuring the transverse voltage in the Hall bar with a 1 mA d.c. read current. In switching protocol II (Fig. S1 B), a single current pulse is used for both writing and simultaneous reading [switching scheme 4 in Fig. S2]. The schematic diagram of four different switching schemes, namely (1) 'ns-write and dc-read' (2) 'µs-write and dc-read' (3) 'ms-write and dc-read', and (4) 'simultaneous read and write', are summarized in Fig. S2.

For scheme 1 ('ns-write and dc-read'), scheme 2 ('µs-write and dc-read'), and scheme 3 ('ms-write and dc-read'), the writing pulses were applied with PSPL10300 (ns pulse with 750 ps rise/fall time), Keithley 4200 (µs pulse with 20 ns rise/fall time), and Keithley 2635B (ms pulse) pulse generators, respectively. The Hall resistance R_{xy} measurements were performed by the Keithley 2635B. For scheme 4, 'simultaneous read and write', a set of Keithley 6221 and 2182a nanovoltmeter, which was trigger-linked, was used. In this scheme, the Hall resistance R_{xy} was measured during the writing current pulse. The diagrams of these measurement sequences are shown in Fig. S1.

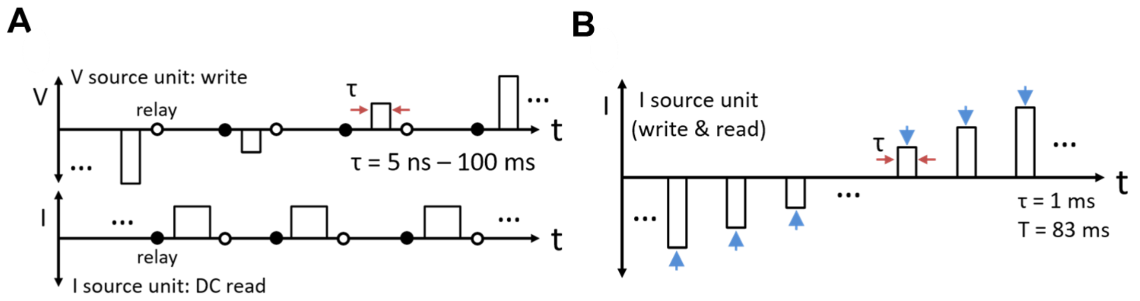

Fig. S1. Current induced Switching protocol. (A) Switching protocol I, where writing pulses of different pulse length (5 ns - 100 ms) were used and R_{xy} was measured after a specific delay with a dc current of 1 mA. For **(A),** internal and external switching relay circuits were used for

switching between writing and reading. Note that open and closed circles indicate open and closed relays, respectively. **(B)** Switching protocol II, where the same current pulses were used for writing and reading. Blue arrows denote the points where the reading data was taken.

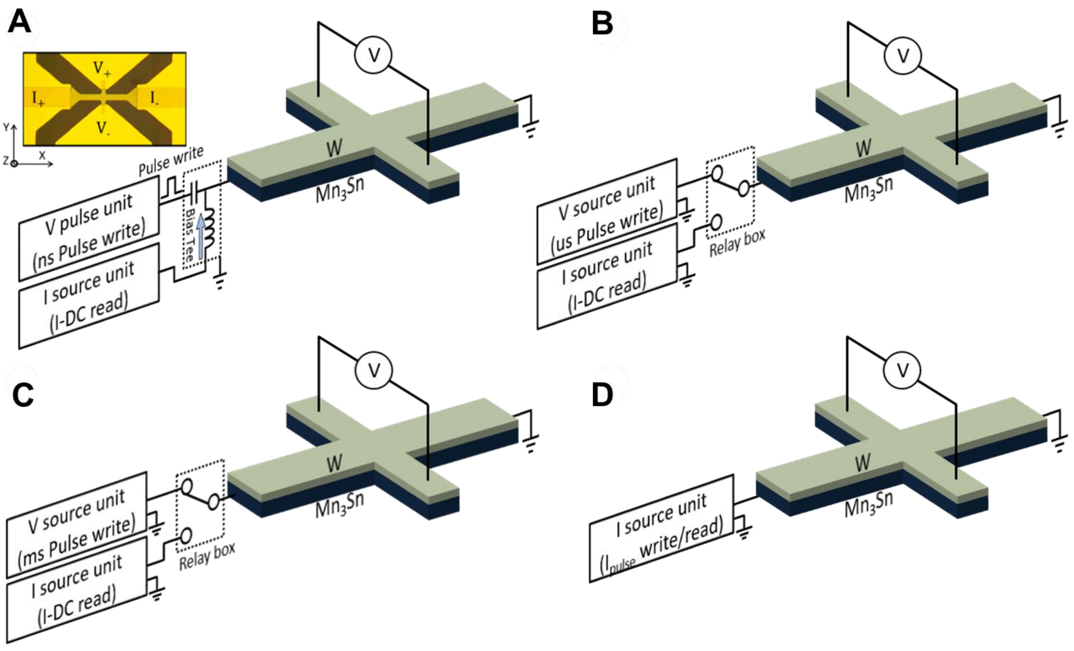

Figure S2. Schematics of different switching. (A) (inset) a microscopic image of a Hall Bar that was used for the switching experiments. **(A)-(C)** Schematics for ns, µs, and ms writing pulse, respectively and delayed reading with dc current (1 mA). **(D)** Schematics for 'simultaneous read and write' measurement. During experiments current was applied along '***x***' and the magnetic field was applied along different direction as mentioned in the text.

I. Structural analysis and magnetic properties of Mn_3Sn thin film

The texture of the polycrystalline Mn_3Sn film was studied by X-ray diffraction using a Bruker D8 Discover four circle diffractometer operated with monochromatized Cu-K-α_1 radiation and a six-circle diffractometer using focused Ga-K-α radiation from a Ga-jet x-ray source.

Fig. S3 a shows the 2θ-θ scan on a logarithmic intensity scale in the range between 2θ = 20° and 80° collected with the Bruker D8 Discover using a two-dimensional pixel detector operated in one-dimensional scan mode. The measurement was carried out on a 100 nm thick Mn_3Sn thin film. Within this angular interval, all reflections could be observed with exception of the (0002) reflection near 2θ = 40° which is too weak, while in other studies it has been detected (29, 47). Already on a qualitative basis this suggests that our film is highly textured with a preferential orientation of the $(11\bar{2}0)$ crystal face parallel to the film surface. Moreover, in our case the $(11\bar{2}0)$ reflection is about three times as strong as compared to the $(20\bar{2}1)$ reflection. The latter should be the strongest one in the ideal case of a polycrystal with a completely random orientation of the crystallites, which is observed in the XRD patterns of Refs (29, 47). The inset shows the reciprocal space map (RSM) in the vicinity of the $(20\bar{2}1)$ reflection. In the RSM data which were collected using a six-circle diffractometer and a Ga-jet X-ray source under grazing incidence (incidence angle μ = 1°) of the primary beam, three diffraction rings are observed which are identified as reflections belonging to TaN $(10\bar{1}1)$ and to the Mn_3Sn (0002) and $(20\bar{2}1)$ reflection. The intensity ratio between the latter is equal to about 1:5. In order to evaluate the presence of a texture in the deposited film we have quantitatively analyzed the reflection intensities by integrating the peaks and deriving an effective multiplicity as shortly outlined as follows:

The integrated intensity [$I(hkil)$] of a polycrystalline (powder) sample at the Bragg angle θ is given by (49)

$$I(hkil) \propto \frac{|F(hkil)|^2 \times \ H \times V_X}{sin^2(\theta)\ \cos(\theta) \times \mu \times v_{uc}^2}$$

where $|F(hkil)|$, V_x, v_{uc} and μ represent the structure factor magnitude, the volume of the crystal, the volume of the unit cell (uc) and the linear absorption coefficient, respectively. Importantly, the factor H represents the multiplicity (i.e. the statistical weight) of the crystal faces in a polycrystalline sample. For instance, in space group (SGR) $P6_3/mmc$ the multiplicity of the basis-pinacoid $\{0001\}$ is equal to 2, while for the hexagonal prism $\{10\bar{1}0\}$ it is equal to 6.

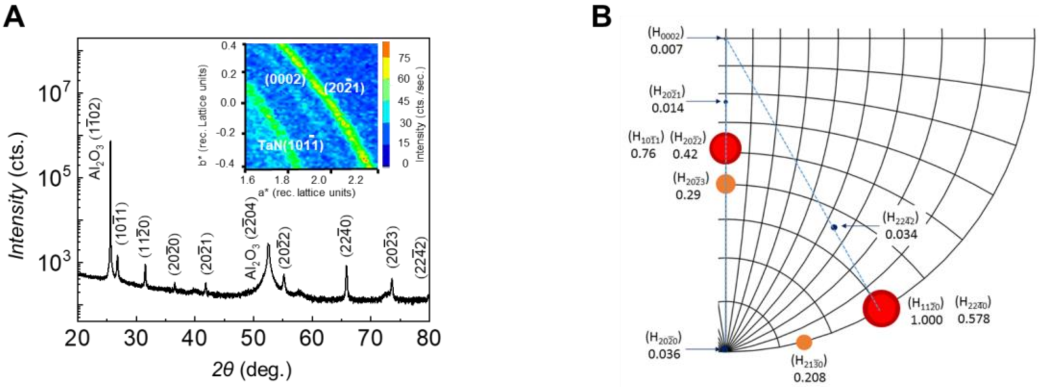

Fig. S3. Crystallographic characterizations of Mn_3Sn. (A) 2θ-θ scan for the Mn_3Sn film on Al_2O_3 $(1\bar{1}02)$. Narrow weak reflections originate from the Mn_3Sn film and are indexed. The inset shows a reciprocal space map in the vicinity of the $(20\bar{2}1)$ reflection. **(B)** Plot of H_{hkil}^{eff} derived for all nine reflections (*hkil*) shown in Fig. S3 (A). The size of the circles approximately represents H_{hkil}^{eff} normalized to 1.0 for the $(11\bar{2}0)$ reflection. Circles are located according to the position of the corresponding crystal faces in a stereographic projection using Wulff's net.

We have used the above equation and calculated values for $|F(hkil)|^2$ to derive the effective H-values (H_{hkil}^{eff}) form the integrated intensities for each reflection (*hkil*). The factors μ, V_{x} and v_{uc} cancel out as we evaluate normalized and relative values for H_{hkil}^{eff}. In an ideal polycrystalline sample (powder) H_{hkil}^{eff} should be equal to 1 for all reflections.

Fig. S3 (B) shows the result, where $H_{hkil}^{\mathrm{eff}}$is roughly represented by the area of the circles. We use a stereographic projection along the c-axis of the crystal lattice to represent the position of the crystal faces. The reflection indices and H_{hkil}^{eff} for all reflections are indicated next to the circles. The most important result is the strong preference of the $(11\bar{2}0)$ reflection while the $(10\bar{1}1)$ reflection also has an appreciable weight. H_{hkil}^{eff} for first and second order reflections deviate by up to 40% which we attribute to both the statistical error resulting from the very weak reflections and to the fact that we are using $|F(hkil)|$ values calculated on the basis of the perfect structure, which is likely not absolutely correct, especially with regard to the presence of disorder. We emphasize however, that a scatter of H_{hkil}^{eff} in the range of 40% is very small as compared to the overall dynamics of H_{hkil}^{eff} throughout the data set. We find small and extremely small values for most reflections [e.g. $(20\bar{2}0)$, (0002), $(20\bar{2}1)$, $(22\bar{4}2)$] which are two orders of magnitude lower than expected for a random orientation of the crystallites. This demonstrates that the sample surface is characterized by a majority (≈ 50%) of crystallites preferentially oriented with their $(11\bar{2}0)$ crystal faces parallel to the sample surface.

Fig. S4 A displays a scanning-TEM micrograph of 30 nm Mn_3Sn film (viewed along $[11\bar{2}0]$ direction) which illustrates that the film is highly ordered locally, although it's textured

globally as found from the XRD analysis. The surface roughness of the film is increased from 2 nm to 4 nm with the increase of film thickness from 30 nm to 100 nm. A typical AFM image for 30 nm Mn_3Sn thin films is shown in Fig. S4 B.

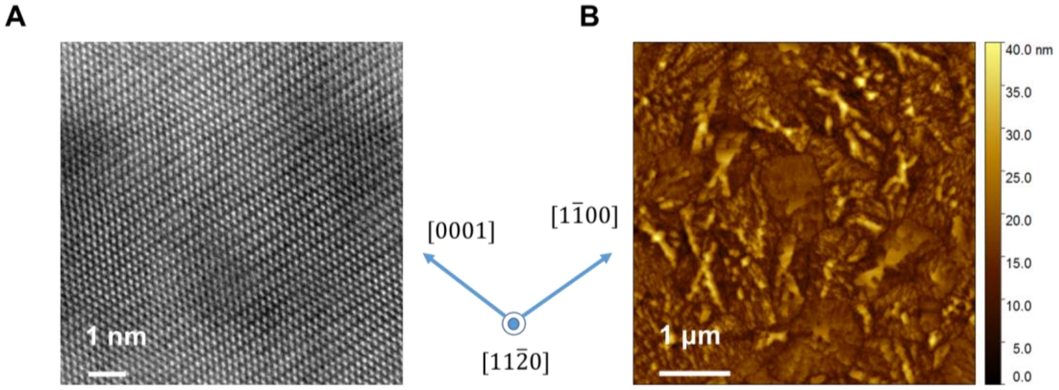

Fig. S4. **STEM and AFM characterization of Mn_3Sn. (A)** Cross-section scanning-TEM (STEM) micrograph of 30 nm Mn_3Sn film which is grown on Al_2O_3 (1$\bar{1}$02) substrate, viewed along the [11$\bar{2}$0] of Mn_3Sn **(B)** Atomic force micrograph of the Mn_3Sn (30 nm) / W (8 nm) bilayer film over 5×5 μm^2 scan area.

To Probe the small moment which lies in the kagome plane of Mn_3Sn, magnetization measurements were carried out as a function of applied out-of-plane magnetic field (H_z ∥ [11$\bar{2}$0]) at T = 300 K). A typical variation of magnetization as a function of H_z is shown in Fig. S5 (A) for Mn_3Sn (80 nm) / W (8 nm) sample where magnetic signal is mostly dominated by a diamagnetic contribution known to originate from the diamagnetic Al_2O_3 substrate. The film, on the other hand, exhibits a very weak magnetic signal. The background diamagnetic moment of the substrate has been subtracted from the raw data and the corrected magnetic data (film contribution) is shown in Fig. S5 (B). The spontaneous magnetization is found to be very small (25 emu/cm^3) as expected for the non-collinear AFM Mn_3Sn thin film (50, 51). The magnetization is almost independent of the film thickness. Further, field cooled (H_{FC} = 10 mT) magnetization measurements have been carried out as function of temperature in the out-of-plane geometry to confirm the inverse triangular structure. It can be seen from Fig. S5 (C) that the magnetization goes through a transition around T = 260 K which manifests the transition from a triangular to a helical structure for the Mn_3Sn film. These results are consistent with previous reports (29, 34).

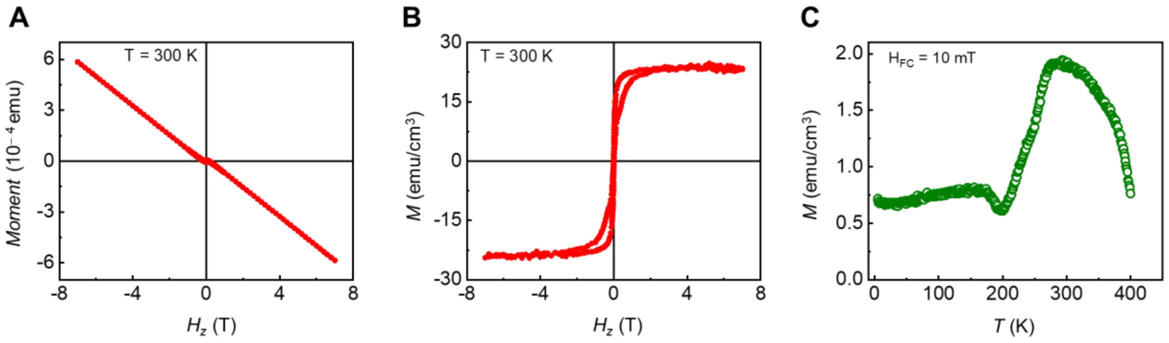

Fig. S5. Magnetic measurements on Mn_3Sn. (A) Magnetic moment as a function of H_z at room temperature (both substrate and thin-film contribution.) **(B)** Magnetization (M) as a function of H_z after substrate contribution is subtracted. **(C)** Temperature dependent evolution of M in field cooled condition ($H_z = 10$ mT).

II. Model tight-binding calculations

The purpose of this section is to show that the net magnetic moment $\boldsymbol{m}$ of a kagome triangle allows to determine the shape of the resistivity tensor, even though it is not the order parameter. For the purpose of generality, we do not restrict our calculations to Mn_3Sn but model a generic kagome magnet that exhibits a phase with a negative vector spin chirality and is AB stacked. Since Mn_3Sn d bands dominate near the Fermi level, we consider 5 d orbitals for each of the three lattice sites giving us 30 bands in total. The electronic Hamiltonian reads

$$H = \sum_{ij\alpha\beta\sigma} t_{\alpha\beta}^{ij} a_{i\alpha\sigma}^{\dagger} a_{j\beta\sigma} + \lambda \sum_{i\alpha\beta\sigma\sigma'} a_{i\alpha\sigma}^{\dagger} \; S \; a_{i\beta\sigma'} + m \sum_{i\alpha\sigma\sigma'} a_{i\alpha\sigma}^{\dagger} \; (\boldsymbol{m}_i \cdot \boldsymbol{\sigma}) \; a_{i\alpha\sigma'}.$$

The first term comprises the hopping terms, accounting for the electrons' kinetic energy. An electron with orbital α and spin σ is annihilated at site i (operator $a_{i\alpha\sigma}^{\dagger}$) and is created with orbital β at site j with the same spin (operator $a_{j\beta\sigma}$). This process is quantified by the hopping amplitude $t_{\alpha\beta}^{ij}$. Note, that not all hopping amplitudes are independent of each other. In fact, for d electrons there exist exactly 3 Slater-Koster parameters to quantify all the hopping amplitudes via a linear combination of directional cosines. We have chosen these Slater-Koster parameters as $V_{\pi} = -V_{\sigma} = -V_{\delta} = 1$ eV. The second term in the electronic Hamiltonian characterizes the spin-orbit coupling with amplitude $\lambda = 0.2$ eV and the interaction matrix

$$S = i \begin{pmatrix} 0 & \sigma_y & -\sigma_x & 2\sigma_z & 0 \\ -\sigma_y & 0 & \sigma_z & -\sigma_x & -\sqrt{3}\sigma_x \\ \sigma_x & -\sigma_z & 0 & -\sigma_y & \sqrt{3}\sigma_y \\ -2\sigma_z & \sigma_x & \sigma_y & 0 & 0 \\ 0 & \sqrt{3}\sigma_x & -\sqrt{3}\sigma_y & 0 & 0 \end{pmatrix},$$

where σ_x, σ_y and σ_z are the Pauli matrices. The SOC term is an onsite term that mixes different orbitals according to S. The third term is the interaction of the electron spin ($\boldsymbol{\sigma}$ vector of Pauli matrices) with the magnetic texture ($\boldsymbol{m}_i$ unit vectors). This is also an onsite term but it mixes different spin directions. The strength of this coupling is $m = 0.2$ eV.

We diagonalize the Hamiltonian to determine the band structure E_n and the eigenvectors $|n\rangle$, both dependent on the reciprocal vector $\boldsymbol{k}$. We calculate the Berry curvature tensor for band *n*

$$\Omega_{n,ij} = -2\ \mathrm{Im} \sum_{m \neq n} \frac{\langle n|\partial_{k_i} H|m\rangle \ \langle m|\partial_{k_j} H|n\rangle}{(E_n - E_m)^2}.$$

From the Berry curvature, we can calculate the intrinsic contribution to the Hall conductivity as the integral over all occupied states (energy below the Fermi energy E_F)

$$\sigma_{ij} = -\frac{e^2}{h}\frac{1}{(2\pi)^2}\sum_n \int_{E_n(\boldsymbol{k})\leq E_F} \Omega_{n,ij}\,(\boldsymbol{k})\ \mathrm{d}\boldsymbol{k}.$$

First, we simulate the six different equilibrium magnetic phases ($\varphi = 30°, 90°, 150°, 210°, 270°, 330°$) that we have discussed in the paper. Our numerical results (three exemplary phases in Fig. S6) reveal that the conductivity tensor can be decomposed as

$$\rho^{-1} = \sigma = \begin{pmatrix} \sigma_{xx} & \sigma_{xy} & \sigma_{xz} \\ \sigma_{yx} & \sigma_{yy} & \sigma_{yz} \\ \sigma_{zx} & \sigma_{zy} & \sigma_{zz} \end{pmatrix} = \sigma_0 \begin{pmatrix} 0 & \sin\varphi & -\cos\varphi \\ -\sin\varphi & 0 & 0 \\ \cos\ \varphi & 0 & 0 \end{pmatrix}.$$

This means, that the plane of Hall transport is perpendicular to the net magnetization, similar to the situation in a ferromagnet.

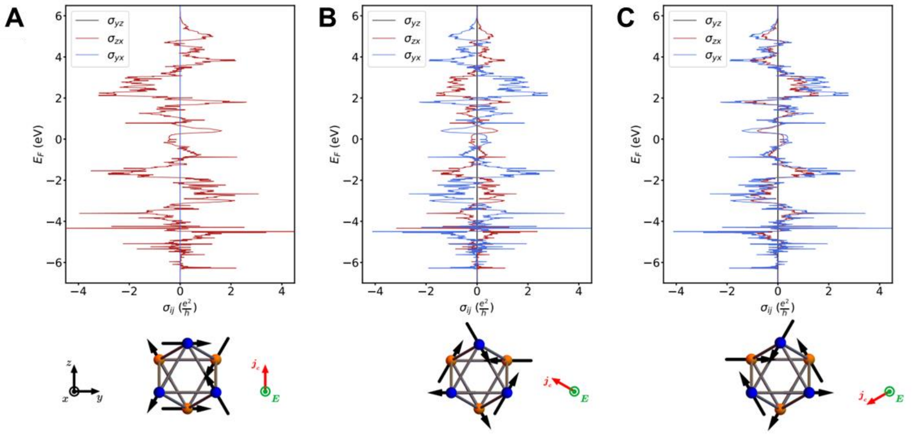

Fig. S6. Hall conductance for different magnetic configurations of Mn3Sn. The kagome plane is the *yz* plane. The corresponding *yz* tensor element is always zero. The other two elements change according to the tensor representation above. The considered configurations in (**A**)-(**C**) correspond to $\varphi = 30°, 90°, 150$ considered in the main text.

However, the mechanism behind it is very different. The net moment is way too small to explain the measured signal. The origin is a set of broken symmetries by the arrangement of magnetic moments. This effect remains, even if we artificially fix the moments at angles 120° with respect to each other, so that the net magnetization is exactly zero. As is shown in Figure S7, this only changes the Hall conductivity very slightly. This means, the net moment is not the order parameter but under the experimental conditions, it still allows to determine the shape of the resistivity tensor. In the experiment, we measure the *xy* element which is why we observe the maximum signal for the field-switching mechanism that involves switching between the $\varphi = 90°$ and $\varphi = 270°$ configurations where the sine has its extrema. For the other four configurations, that are relevant for the SOT switching, the sine is only ± 0.5.

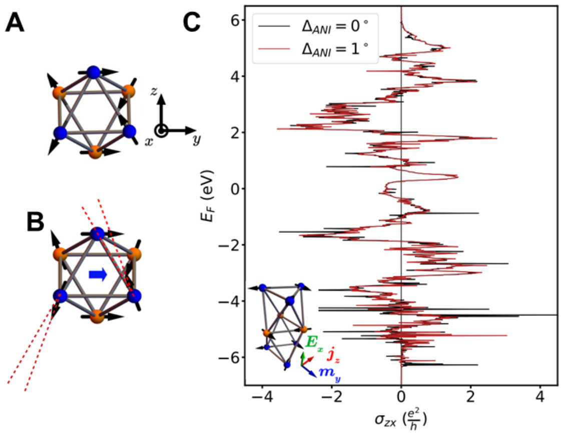

Fig. S7. Hall conductance with and without net magnetic moment. (**A,B**) show stable magnetic configurations in Mn3Sn without and with easy-axis anisotropy taken into account

[dashed lines in (**B**); effect exaggerated]. The configuration in (**A**) has no net moment, $\boldsymbol{m} = 0$. In (**C**) the Hall conductivity is shown for both configurations. The difference is only marginal.

III. Theoretical simulation of current induced switching

We have carried out theoretical simulation of current induced switching as discussed in the previous publication (29). When a current is applied along $\boldsymbol{x}$, the SHE in W injects a spin current with a polarization $\boldsymbol{s} \parallel \pm \boldsymbol{y}$ into the Mn_3Sn layer, *i.e.*, along $\varphi = 0°$ or $\varphi = 180°$, depending on the sign of the current. If the current density is sufficiently large, the injected spin current orients all the magnetic configurations along these two unstable configurations (cf. energy maxima in Fig. 1C in the main text). Using atomistic simulations this change in the magnetic state can be calculated, as illustrated in Fig. S8 (A, B). The absence of the bias field (H_x) leads to a perfect alignment of $\boldsymbol{m}$ along the two directions characterized by $\varphi = 0°, 180°$, once the applied current is larger than the critical current. These configurations correspond to two energy maxima shown in Fig. 1C. For this reason, once the current is turned off, domains in the configuration $\varphi = 0°$ will relax either to $\varphi = 30°$ or $\varphi = 330°$ (two equally close energy minima). The probability for both relaxation processes is equal and since the two configurations are characterized by $m_z = +0.5m$ and $m_z = -0.5m$, respectively, the measured xy Hall resistivity cancels. The same argument holds for the domains that are in a $\varphi = 180°$ state. These relax either to $\varphi = 150°$ or $\varphi = 210°$.

In the presence of H_x, the metastable states never reach exactly the injected spin orientations $\varphi = 0°$ and $\varphi = 180°$ but approach close to these values depending on the current density. The direction (sign with respect to **z**) of $\boldsymbol{H} \times \boldsymbol{s}$ determines whether φ is slightly smaller or larger than these saturation values. When the current is turned off, the system then relaxes to the nearest energy minimum depending on the sign of $\boldsymbol{H} \times \boldsymbol{s}$. This means that by reversing the current direction (and therefore $\boldsymbol{s}$), the magnetic configurations are switched from either $\varphi = 330°$ to $30°$ or from $\varphi = 210°$ to $150°$ and vice-versa (depending on the H_x direction), thereby changing the sign of the Hall voltage. For both metastable states, this corresponds to a switching between configurations with a projected net moment of $m_z = \pm 0.5 |\boldsymbol{m}|$. Therefore, the theoretically highest possible Hall signal is half as large as that in the field-switching experiment, i.e., $\sim \pm 25$ mΩ for the present study.

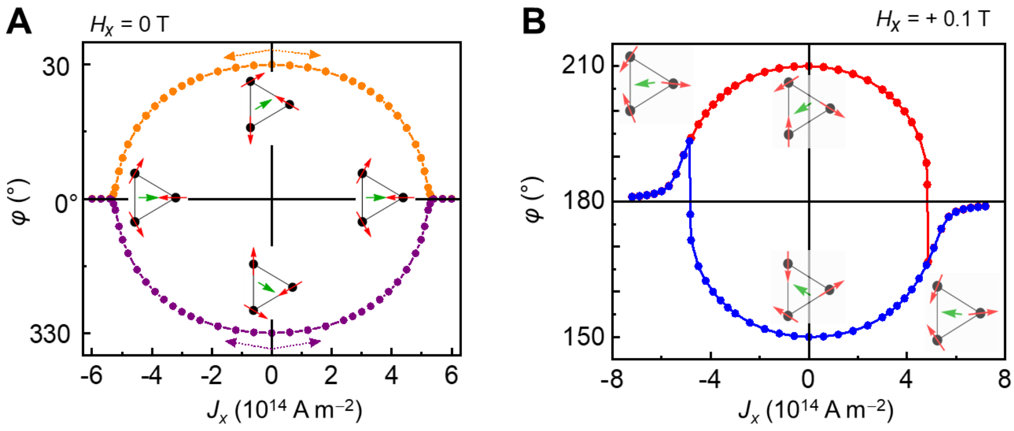

Fig. S8. Theoretical simulation of electrical switching. (A)-(B) Theoretical description of current-induced SOT switching in the presence of a bias field H_x = 0 T and 0.1 T.

IV. Determination of device temperature from current induced switching using Protocol II

Current induced switching experiment is shown in Fig. S9 A using Protocol II in the absence of bias field (H_x=0). The non-linear behavior of R_{xy} reflects that a finite R_{xx} contribution to R_{xy} due to a small mismatch in the voltage contacts and this helps to calibrate the device temperature. Taking the R_{xy} value at J_x = 0 for zero field at different measurement temperature (inset of Fig. S9 A), the device temperature is found to be 435 K when switching occurs. The dependence of J_c on the pulse length and as a function of temperature, is summarized in Fig. S9 B (from Protocol II).

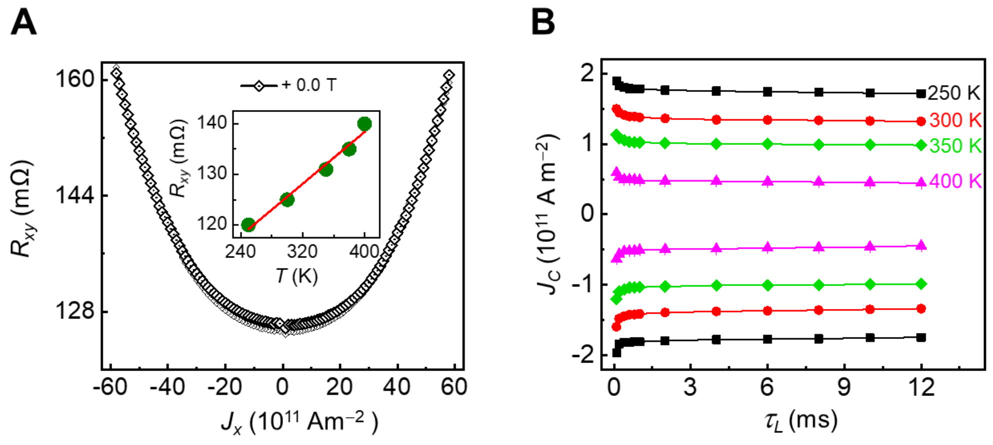

Fig. S9. Device temperature from protocol II and pulse width dependent critical current variation. (A) R_{xy} as a function of J_x with H_x = 0 T using Switching protocol II. In inset R_{xy} value at J_x = 0 is plotted against measurement temperature to calibrate the device temperature **(B)** Dependence of J_c on current pulse length and measurement temperature.

V. Magneto-transport properties of Mn_3Sn thin films

Fig. S10 A shows the zero-field longitudinal resistivity (ρ_{xx}) as a function of temperature over the temperature range from 5K to 400K for the 30 nm Mn_3Sn thin film. It exhibits a metallic transport behavior as expected with residual resistivity ratio (RRR) ~ 1.75 which demonstrates a high quality of thin film. ρ_{xx} is 180±10 μΩ cm at room temperature for the different thicknesses of Mn_3Sn. We have also measured the longitudinal resistance (R_{xx}) of the device during the writing pulse application. Lower inset of Fig. S10 A shows that the resistance of the device is increasing more than 12 Ω for a write pulse of magnitude ±9V. By comparing this change in R_{xx} with temperature dependent R_{xx} measurements (upper inset of Fig. S10 A) we conclude that the temperature of the device is beyond 400 K for a voltage close to ±9V. Variable temperature Hall measurements were carried out to investigate the change in Hall signal when the temperature of the system is increased. A few representative R'_{xy} vs H_z plots at different temperatures for the Mn_3Sn (30 nm) / W (8 nm) bi-layer are shown in Fig. S10 B. It can be seen that R'_{xy} for $H = 0$ exhibits a large change from 50 to -50 mΩ at $T = 300$ K. The change in the zero field Hall resistance (R'_{xy}at $H_z = 0$) and coercivity (H_c) are plotted against temperature as depicted in Fig. S10 C.

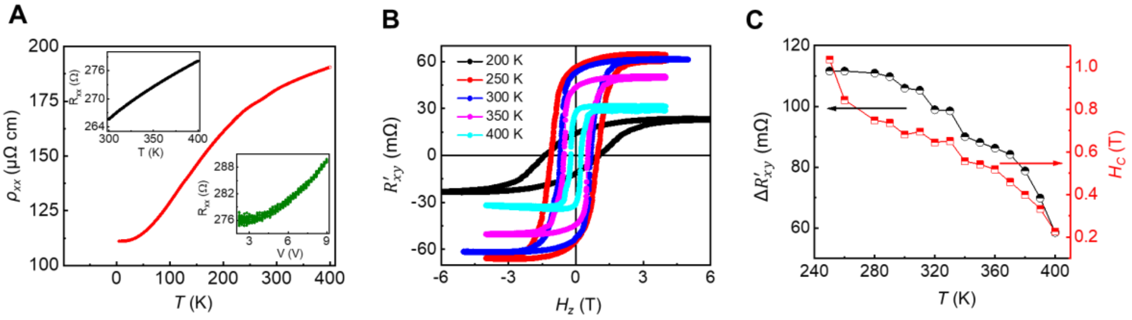

Fig. S10. Longitudinal resistivity and anomalous Hall measurements. (A) Variation of ρ_{xx} as a function of temperature at $H_z = 0$ T. Upper inset shows actual resistance of the device as a function of temperature. Lower inset shows the change of device resistance during writing pulse sweep. **(B)** H_z dependence ofR'_{xy} at different temperatures. **(C)** Temperature dependence of $\Delta R'_{xy}$ which is extracted by measuring anomalous Hall hysteresis at different temperatures.

VI. Thermal randomization in Mn_3Sn in absence of spin current

The anomalous Hall and current induced switching of single layer 40 nm Mn_3Sn is shown in Fig. S11 A and B. Although there is no spin current source, R'_{xy} rapidly decreases at a critical current. This clearly show that there is no role of spin orbit torque for the setting of magnetic states or chiral spin rotation (30) rather the magnetic states sets by thermally.

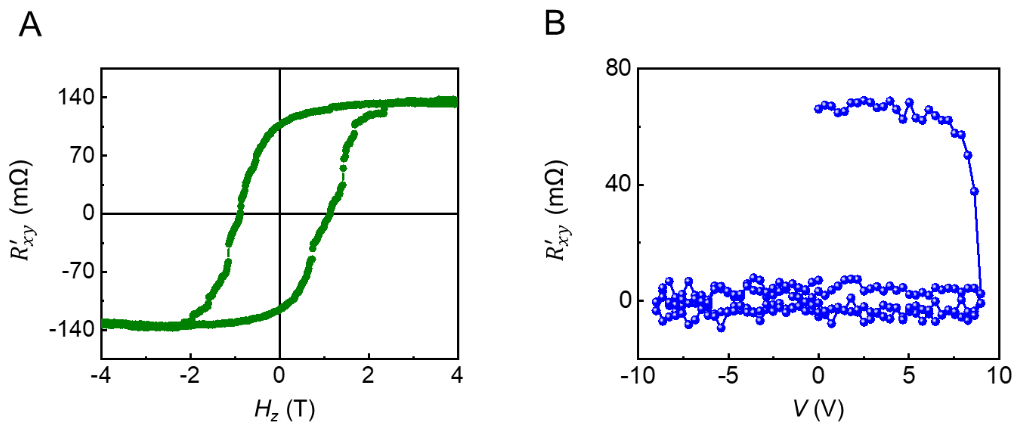

Fig. S11. Anomalous Hall resistance and electrical switching of Mn_3Sn without W. (A) R'_{xy} as a function of H_z at room temperature for 40 nm single layer Mn_3Sn. **(B)** Current-induced switching with H_x = 0.0 T for the same sample.

VII. Current-induced switching of different thicknesses of Mn_3Sn/W bi-layers

Here, we show the current-induced switching of the other prepared thicknesses of Mn_3Sn (d_{AF} = 40, 60, 80 and 100 nm) with a fixed thickness of 8 nm of W. The change in Hall resistance ($\Delta R'_{xy}$) during the magnetic-field-induced switching is shown in Fig. S12-15 A for the 40 to100 nm Mn_3Sn thin films. The corresponding electrical switching, based on the type-I protocol, with H_x = ± 0.1 T, is displayed in Fig. S12-15 B. It is evident that the threshold current density is the same for all thicknesses and percentage of switching is ~ 40 % for all films except 100 nm film.

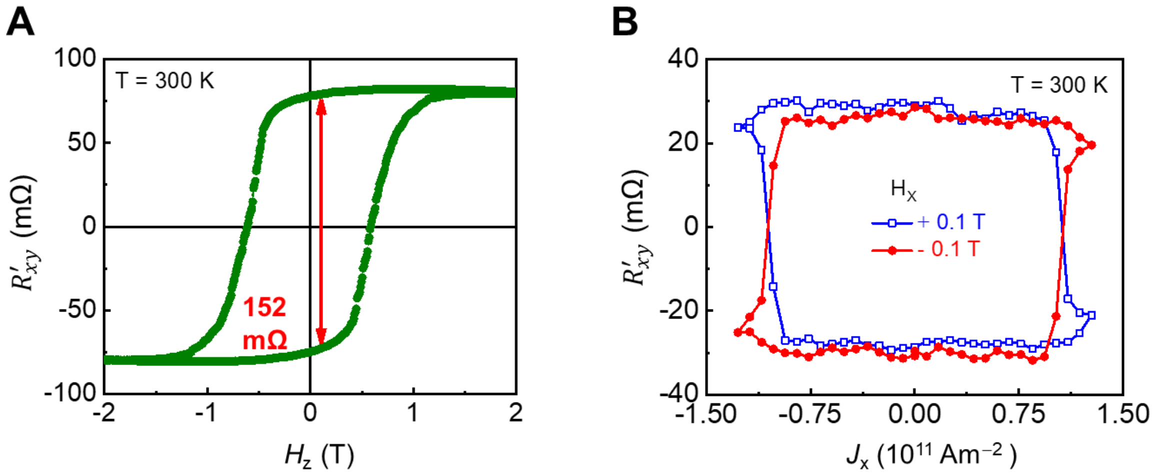

Fig. S12. Anomalous Hall resistance and electrical switching of 30 nm Mn_3Sn. **(A)** R'_{xy} as a function of H_z at room temperature. **(B)** Current-induced switching with H_x = ± 0.1T.

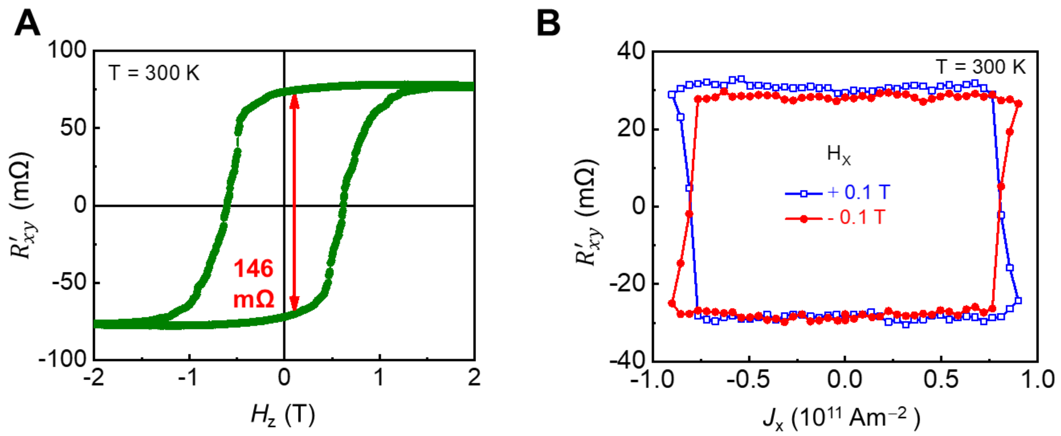

Fig. S13. Anomalous Hall resistance and electrical switching of 60 nm Mn_3Sn. (A) R'_{xy} as a function of H_z at room temperature. **(B)** Current-induced switching with $H_x = \pm 0.1$T.

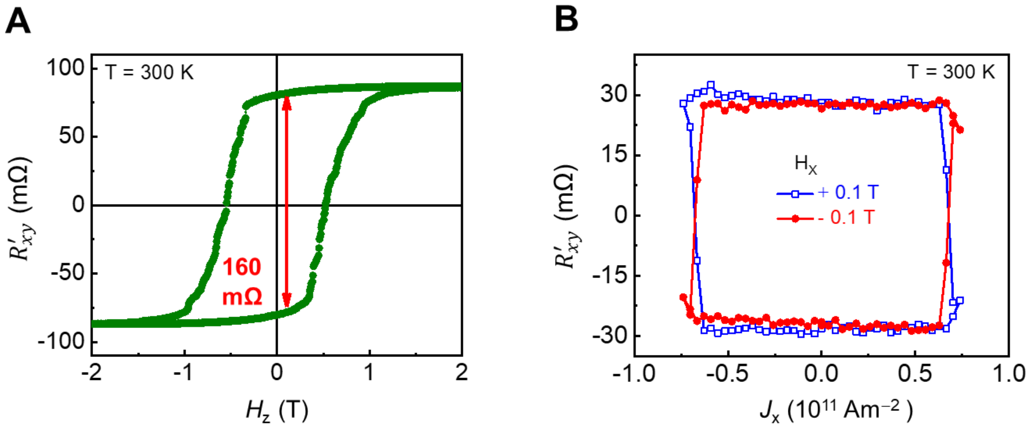

Fig. S14. Anomalous Hall resistance and electrical switching of 80 nm Mn_3Sn. (A) R'_{xy} as a function of H_z at room temperature. **(B)** Current-induced switching with $H_x = \pm 0.1$ T.

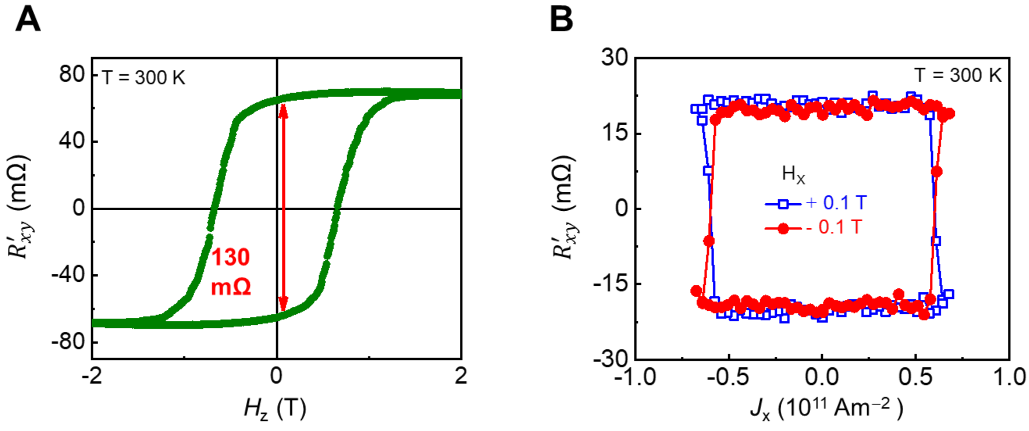

Fig. S15. Anomalous Hall resistance and electrical switching of 100 nm Mn_3Sn/W/TaN. (A) R'_{xy} as a function of H_z at 300 K. **(B)** Current-induced switching with $H_x = \pm 0.1$T.

VIII. Numerical modeling of the Joule heating and cooling during current pulse application

Here we present the details of the finite element method for the Joule heating and cooling simulations that were used to estimate the variation of the device temperature during the current-induced switching. The 'Joule heating' and the 'heat transfer' modules of the COMSOL multi-physics software were used for this study. First, a rectangular writing pulse (with amplitude V_0 and pulse length, τ_L) were applied on the Hall bar device (Fig. S16). The time (t) dependent evolution of the temperature (T) during and after application of the writing pulse was estimated by numerically solving the transient heat-diffusion equation:

$$\rho C_p \frac{\partial T}{\partial t} = \kappa \nabla^2 T + \frac{J^2}{\sigma}$$

where ρ is the density, C_p is the specific heat capacity, T is the temperature, κ is the thermal heat conductivity, J is the applied current density and σ is electrical conductivity of the device. Since all our measurements were carried out in vacuum condition, convective cooling processes were not included in our simulation. The Hall bar of our experiment is comprised of a stack sequence Al_2O_3 (substrate) / Mn_3Sn (30 nm) / W (8 nm) / TaN (3 nm). Here, in COMSOL, this Hall bar is modeled as a single layer metallic film whose effective electrical conductivity (σ) and thermal conductivity (κ) were estimated from the σ and κ of Mn_3Sn and W. Since these devices consist mainly of the Mn_3Sn layer, other materials properties were approximated for Mn_3Sn only.

Fig. S16 A shows a representative temperature profile of a device during the application of a writing pulse as obtained from the simulation. The color plot (Fig. S16 A and C) shows that the device heats up significantly compared to its surrounding. For a better estimation, the time dependent variation of the temperature of the device, as obtained from a point in the center of the Hall bar, is shown in Fig. S16 B for the application of a square-shaped writing pulse (τ_L ~100 ns) of different magnitudes (V_0). Two important observations can be made from these data as explained in the main section: (1) Depending on the magnitude of the writing pulse, the device temperature can increase significantly even for a 100 ns pulse. (2) The system cools down relatively fast if the applied writing pulse is switched off on a sub-ns time scale. In particular, the temperature drop is approximately 30% of the maximum temperature after 20 ns $\left(\frac{\Delta T_{100\ ns} - \Delta T_{120\ ns}}{\Delta T_{100\ ns}}\right) \times 100\% \approx 30\%$).

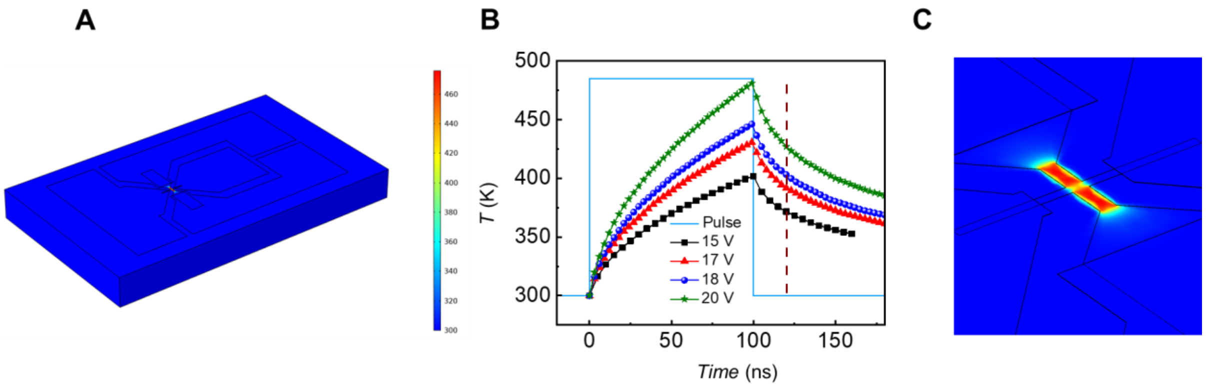

Fig. S16. Joule heating and cooling simulations using COMSOL. (A) Geometry of the sample used for the simulation. **(B)** Variation of the device temperature under the application of a 100 ns pulse with different applied voltages. **(C)** Closer look of the device during current pulse application.

IX. Estimation of ΔR_{xy} and J_c during H_x dependent switching experiments

In Fig. 5, B and C of the main text (for Mn_3Sn), $\Delta R'_{xy}$ and J_c were estimated in four different conditions. $\Delta R'_{xy}$ are defined as $\Delta R'_{xy} = [R'_{xy}\ (\text{after switch}) - R'_{xy}\ (\text{before switch})]$

(1) Black points ($+H_x$ and $-J_x$) in Fig. 5, A and C: Estimated when H_x is positive and the current sweep is from zero to the negative direction as shown here in Fig. S17, A. The value of $\Delta R'_{xy}$ is positive in this case.

(2) Red points ($+H_x$ and $+J_x$) in Fig. 5, B and C: Estimated when H_x is positive and the current sweep is from zero to the positive direction as shown here in Fig. S17, B. The value of $\Delta R'_{xy}$ is negative in this case.

(3) Purple points ($-H_x$ and $-J_x$) in Fig. 5, B and C: Estimated when H_x is negative and the current sweep is from zero to the negative direction as shown in here in Fig. S17, C. The value of $\Delta R'_{xy}$ is negative in this case.

(4) Blue points ($-H_x$ and $+J_x$) in Fig. 5, B and C: Estimated when H_x is negative and the current sweep is from zero to the positive direction as shown in here in Fig. S17, D. The value of $\Delta R'_{xy}$ is positive in this case.

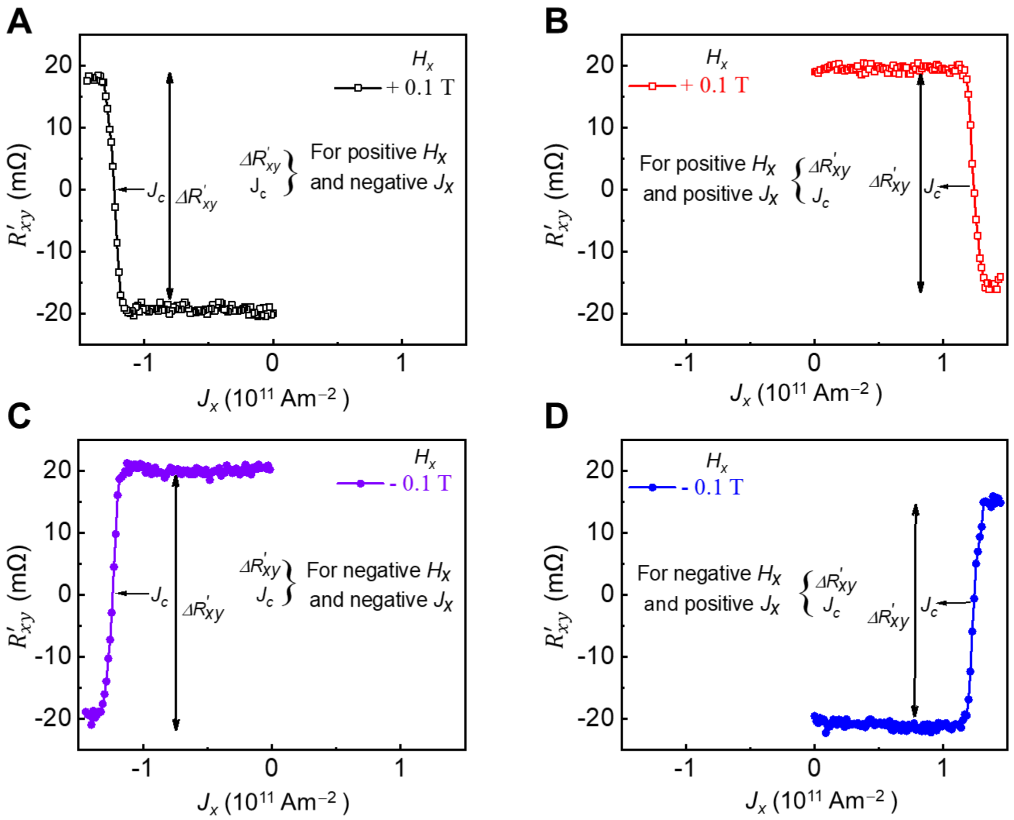

Fig. S17. Estimation of ΔR_{xy} and J_c from electrical switching. Four different cases, (**A**) positive H_x and negative J_x (**B**) positive H_x and positive J_x (**C**) negative H_x and negative J_x (**D**) negative H_x and positive J_x.

X. Switching mechanism comparison with standard FM switching in a CoFeB film

Here we compare the switching mechanism of Mn_3Sn with the standard switching mechanism of a perpendicular magnetic anisotropic (PMA) ferromagnet Ta (5 nm) / CoFeB (1 nm) / MgO (2 nm) /Ta (3 nm). Results of current-induced switching (protocol-I) at different H_x for the PMA ferromagnet are summarized in Fig. S18. In the case of CoFeB, J_c shows a strong dependence on H_x (Fig. S18 B). It decreases monotonically with increasing H_x. However, in case of Mn_3Sn, J_c does not show any dependence on the applied H_x (Fig. 5B, main text). This is because the field only serves as a bias and becomes only relevant when the critical current is reached and the system is in a switchable state. Also, in the case of the normal PMA sample $\Delta R'_{xy}$ is almost independent of the applied H_x (Fig. S18 C). This is because the switching happens always between two distinct magnetic states. Intriguingly, $\Delta R'_{xy}$ in Mn_3Sn exhibits a strong non-monotonic dependence on the applied magnetic field as is evident from Fig. 5C in the main text.

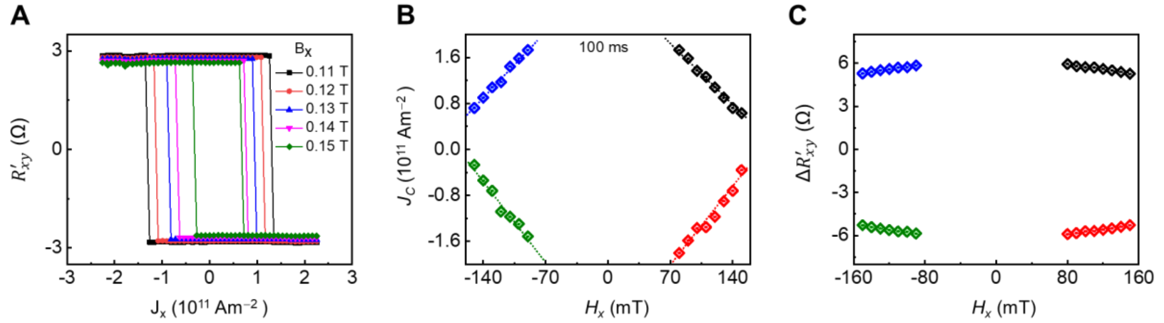

Fig. S18. Electrical switching of PMA CoFeB film. (A) Current-induced switching using different H_x. (**B, C**) H_x dependence of J_c and $\Delta R'_{xy}$.

XI. Combination of field and current induced switching

In the main manuscript, we have discussed two distinct switching mechanisms: A field switching mechanism, when the field H_z is oriented along $\varphi=\pm\ 90°$, and a SSOT switching, for which a field perpendicular to the kagome plane (H_x) provides a bias $\Delta\varphi$ during the switching. Next, we explore the combination of both effects. We apply a field of ±100 mT at an angle ψ with respect to $\boldsymbol{x}$ and within the xz plane (Fig. S19 A). A very interesting finding is that there are now 4 distinct R'_{xy} states whose magnitudes are highly sensitive to ψ as shown in Fig. S19 B. At $\psi = 0°$, R'_{xy} = ±20 mΩ is measured; the same value as discussed earlier for H oriented along $\pm x$. Once H is tilted away from x, we observe that there are two branches for R'_{xy} corresponding to ±H, shown in Fig. S19 B as blue and red. For each ψ along each branch, the sample can be switched hysteretically by current, between two distinct R'_{xy} states, whose difference decreases with increasing ψ. The average values of these states are shifted either upwards (blue) and downwards (red) from zero, as ψ is varied away from zero. R'_{xy} can also be switched from the red to the blue hysteresis curve when the field is reversed but only if the critical current is exceeded. In particular, for $\psi = 90°$, the maximum values of R'_{xy} in each of the branches approaches those values obtained in pure magnetic field switching i.e. ±50 mΩ (see Fig. S10 B). However, here a field of just 100 mT is required as compared to 1T needed for pure field switching. The difference is caused by the heating provided by the current. Note that for $\psi = 90°$ the SOT provided by the current has no effect on the switching and, therefore, cannot provide a seeding layer. Still, it heats up the sample and the magnetic field (H_z along $\varphi = \pm 90°$) provides the bias throughout the whole Mn_3Sn layer. In short, the field-switching mechanism is also strongly affected by current-induced heating in a favorable way. Finally, Fig. S19 C shows how the 4 states can be accessed for $\psi = 10°$ by

applying current pulses in the presence of $H = \pm 0.1$ T. We conclude that there are 4 distinct R'_{xy} states that can be accessed by a current-induced switching mechanism that is strongly influenced by relatively small magnetic fields. These 4 states do not correspond to four individual magnetic phases but to different ratios of the six energetically preferred states presented in Fig. 1C in the main manuscript.

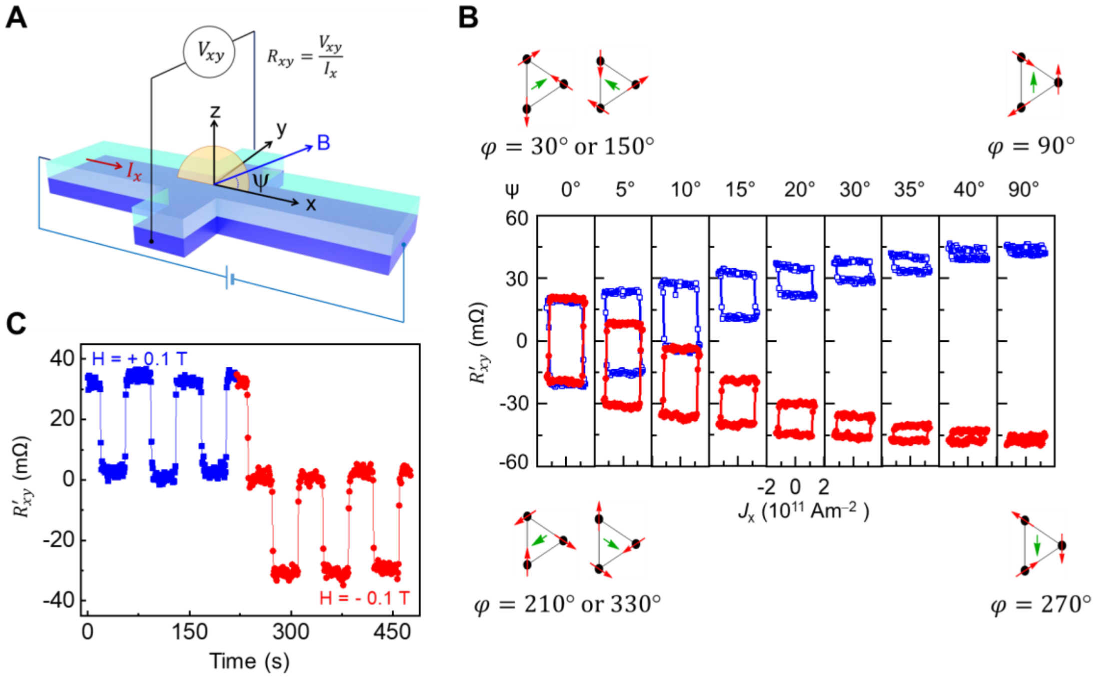

Fig. S19. Combination of field and current induced switching. (A) Schematic of measurement protocol. **(B)** R'_{xy} as a function of ψ in the *xz* plane**. (C)** Switching from positive *H* (+0.1 T) hysteretic loop to negative *H* (-0.1 T) hysteretic loop for ψ = 10°.

XII. Fast Magnetization reversal with nanosecond current pulse in presence of a bias

SSOT mediated switching can only take place if the device relaxes from the ordering temperature in presence of SOT provided by a critical spin current density (S^*). Switching experiments using nanosecond current pulses (with fall time ~ 750 ps) lead to very small switching efficiency (ξ) since the spin current source turnoff abruptly even before the system relaxes below T^*. Therefore to overcome this situation, switching experiments were carried out using nanosecond current pulses ranging from 100 ns to 10 ns in presence of a bias (Fig. S20). It is to be noted that the role of the bias is to provide critical spin current density when the system (S^*) when the system cools below T^*. We note that the magnitude of the bias

voltage (2V) is below the critical current density necessary to switch the device.

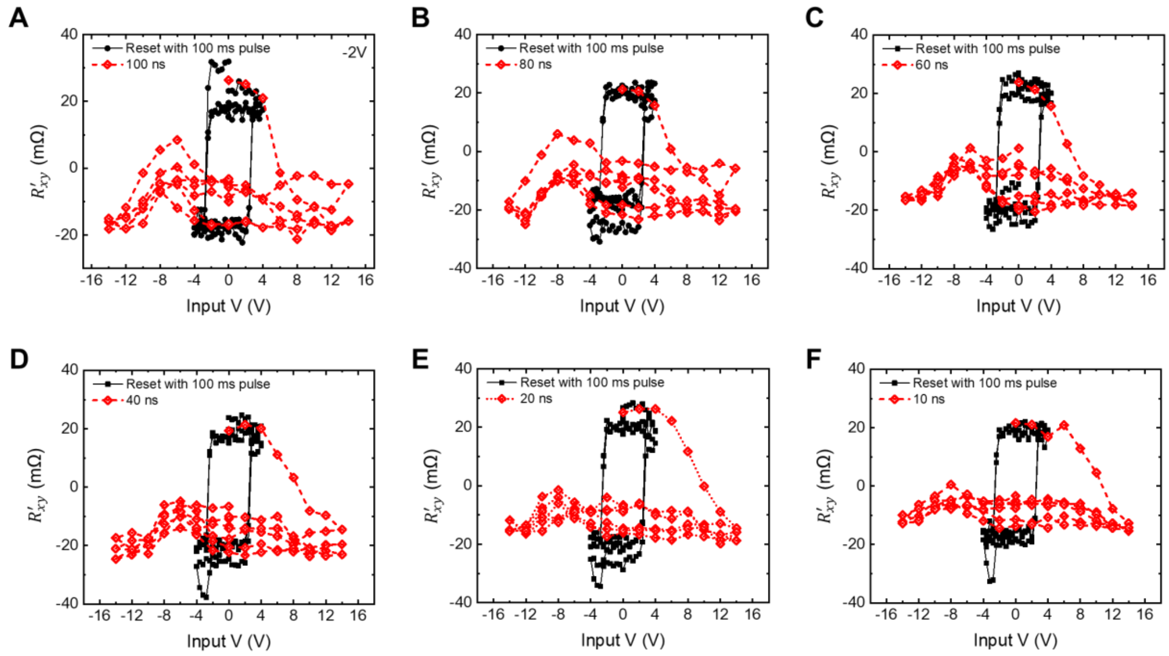

Fig. S20. Electrical switching with ns pulse in presence of dc bias. (A) - (F), Switching experiments using nanosecond current pulses ranging from 100 ns to 10 ns in presence of a bias (2V).

REFERENCES AND NOTES

1. A. Brataas, A. D. Kent, H. Ohno, Current-induced torques in magnetic materials. *Nat. Mater.* **11**, 372–381 (2012).

2. A. Manchon, J. Železný, I. M. Miron, T. Jungwirth, J. Sinova, A. Thiaville, K. Garello, P. Gambardella, Current-induced spin-orbit torques in ferromagnetic and antiferromagnetic systems. *Rev. Mod. Phys.* **91**, 035004 (2019).

3. I. M. Miron, K. Garello, G. Gaudin, P.J. Zermatten, M. V. Costache, S. Auffret, S. Bandiera, B. Rodmacq, A. Schuhl, P. Gambardella, Perpendicular switching of a single ferromagnetic layer induced by in-plane current injection. *Nature* **476**, 189–193 (2011).

4. K. Ando, S. Takahashi, K. Harii, K. Sasage, J. Ieda, S. Maekawa, E. Saitoh, Electric manipulation of spin relaxation using the spin Hall effect. *Phys. Rev. Lett.* **101**, 036601 (2008).

5. A. Chernyshov, M. Overby, X. Liu, J.K. Furdyna, Y. Lyanda-Geller, L.P. Rokhinson, Evidence for reversible control of magnetization in a ferromagnetic material by means of spin–orbit magnetic field. *Nat. Phys.* **5**, 656–659 (2009).

6. L. Liu, T. Moriyama, D. C. Ralph, R. A. Buhrman, Spin-torque ferromagnetic resonance induced by the spin Hall effect. *Phys. Rev. Lett.* **106**, 036601 (2011).

7. I. Mihai Miron, G. Gaudin, S. Auffret, B. Rodmacq, A. Schuhl, S. Pizzini, J. Vogel, P. Gambardella, Current-driven spin torque induced by the Rashba effect in a ferromagnetic metal layer. *Nat. Mater.* **9**, 230–234 (2010).

8. J. Yu, D. Bang, R. Mishra, R. Ramaswamy, J. H. Oh, H. J. Park, Y. Jeong, P. van Thach, D. K. Lee, G. Go, S. W. Lee, Y. Wang, S. Shi, X. Qiu, H. Awano, K. J. Lee, H. Yang, Long spin coherence length and bulk-like spin–Orbit torque in ferrimagnetic multilayers. *Nat. Mater.* **18**, 29–34 (2019).

9. T. Jungwirth, X. Marti, P. Wadley, J. Wunderlich, Antiferromagnetic spintronics. *Nat. Nanotechnol.* **11**, 231–241 (2016).

10. V. Baltz, A. Manchon, M. Tsoi, T. Moriyama, T. Ono, Y. Tserkovnyak, Antiferromagnetic spintronics. *Rev. Mod. Phys.* **90**, 015005 (2018).

11. P. Wadley, B. Howells, J. Železný, C. Andrews, V. Hills, R. P. Campion, V. Novák, K. Olejník, F. Maccherozzi, S. S. Dhesi, S. Y. Martin, T. Wagner, J. Wunderlich, F. Freimuth, Y. Mokrousov, J. Kuneš, J. S. Chauhan, M. J. Grzybowski, A. W. Rushforth, K. W. Edmonds, B. L. Gallagher, T. Jungwirth, Electrical switching of an antiferromagnet. *Science* **351**, 587–590 (2016).

12. J. Železný, H. Gao, K. Výborný, J. Zemen, J. Mašek, A. Manchon, J. Wunderlich, J. Sinova, T. Jungwirth, Relativistic néel-order fields induced by electrical current in antiferromagnets. *Phys. Rev. Lett.* **113**, 157201 (2014).

13. L. Šmejkal, J. Železný, J. Sinova, T. Jungwirth, Electric control of dirac quasiparticles by spin-orbit torque in an antiferromagnet. *Phys. Rev. Lett.* **118**, 106402 (2017).

14. Y. Cheng, S. Yu, M. Zhu, J. Hwang, F. Yang, Electrical r in $\alpha{-}Fe_2O_3$ epitaxial films. *Phys. Rev. Lett.* **124**, 027202 (2020).

15. X. Z. Chen, R. Zarzuela, J. Zhang, C. Song, X. F. Zhou, G. Y. Shi, F. Li, H. A. Zhou, W. J. Jiang, F. Pan, Y. Tserkovnyak, Antidamping-torque-induced switching in biaxial antiferromagnetic insulators. *Phys. Rev. Lett.* **120**, 207204 (2018).

16. M. Dunz, T. Matalla-Wagner, M. Meinert, Spin-orbit torque induced electrical switching of antiferromagnetic MnN. *Phys. Rev. Research* **2**, 013347 (2020).

17. S. Y. Bodnar, L. Šmejkal, I. Turek, T. Jungwirth, O. Gomonay, J. Sinova, A. A. Sapozhnik, H.J. Elmers, M. Kläui, M. Jourdan, Writing and reading antiferromagnetic Mn_2Au by Néel spin-orbit torques and large anisotropic magnetoresistance. *Nat. Commun.* **9**, 348 (2018).

18. T. Hajiri, S. Ishino, K. Matsuura, H. Asano, Electrical current switching of the noncollinear antiferromagnet Mn_3GaN. *Appl. Phys. Lett.* **115**, 052403 (2019).

19. T. Hajiri, K. Matsuura, K. Sonoda, E. Tanaka, K. Ueda, H. Asano, Spin-orbit-torque switching of noncollinear antiferromagnetic antiperovskite manganese NitrideMn3GaN. *Phys. Rev. Appl.* **16**, 024003 (2021).

20. S.-H. Yang, R. Naaman, Y. Paltiel, S. S. P. Parkin, Chiral spintronics. *Nat. Rev. Phys.* **3**, 328–343 (2021).

21. M. Meinert, D. Graulich, T. Matalla-Wagner, Electrical switching of antiferromagnetic Mn_2Au and the role of thermal activation. *Phys. Rev. Appl.* **9**, 064040 (2018).

22. C. C. Chiang, S. Y. Huang, D. Qu, P. H. Wu, C. L. Chien, Absence of evidence of electrical switching of the antiferromagnetic néel vector. *Phys. Rev. Lett.* **123**, 227203 (2019).

23. W. Zhang, W. Han, S.H. Yang, Y. Sun, Y. Zhang, B. Yan, S. S. P. Parkin, Giant facet-dependent spin-orbit torque and spin Hall conductivity in the triangular antiferromagnet $IrMn_3$. *Sci. Adv.* **2**, e1600759 (2016).

24. A. K. Nayak, J. E. Fischer, Y. Sun, B. Yan, J. Karel, A. C. Komarek, C. Shekhar, N. Kumar, W. Schnelle, J. Kübler, C. Felser, S. S. P. Parkin, Large anomalous Hall effect driven by a nonvanishing Berry curvature in the noncolinear antiferromagnet Mn_3Ge. *Sci. Adv.* **2**, e1501870 (2016).

25. S. Nakatsuji, N. Kiyohara, T. Higo, Large anomalous Hall effect in a non-collinear antiferromagnet at room temperature. *Nature* **527**, 212–215 (2015).

26. H. Yang, Y. Sun, Y. Zhang, W.J. Shi, S. S. P. Parkin, B. Yan, Topological weyl semimetals in the chiral antiferromagnetic materials Mn_3Ge and Mn_3Sn. *New J. Phys.* **19**, 015008 (2017).

27. T. Higo, H. Man, D. B. Gopman, L. Wu, T. Koretsune, O. M. J. van 't Erve, Y. P. Kabanov, D. Rees, Y. Li, M.T. Suzuki, S. Patankar, M. Ikhlas, C. L. Chien, R. Arita, R. D. Shull, J. Orenstein, S. Nakatsuji, Large magneto-optical Kerr effect and imaging of magnetic octupole domains in an antiferromagnetic metal. *Nat. Photonics* **12**, 73–78 (2018).

28. A. L. Balk, N. H. Sung, S. M. Thomas, P. F. S. Rosa, R. D. McDonald, J. D. Thompson, E. D. Bauer, F. Ronning, S. A. Crooker, Comparing the anomalous Hall effect and the magneto-optical

Kerr effect through antiferromagnetic phase transitions in Mn_3Sn. *Appl. Phys. Lett.* **114**, 032401 (2019).

29. H. Tsai, T. Higo, K. Kondou, T. Nomoto, A. Sakai, A. Kobayashi, T. Nakano, K. Yakushiji, R. Arita, S. Miwa, Y. Otani, S. Nakatsuji, Electrical manipulation of a topological antiferromagnetic state. *Nature* **580**, 608–613 (2020).

30. Y. Takeuchi, Y. Yamane, J. Y. Yoon, R. Itoh, B. Jinnai, S. Kanai, J. Ieda, S. Fukami, H. Ohno, Chiral-spin rotation of non-collinear antiferromagnet by spin–orbit torque. *Nat. Mater.* **20**, 1364–1370 (2021).

31. P. K. Muduli, T. Higo, T. Nishikawa, D. Qu, H. Isshiki, K. Kondou, D. Nishio-Hamane, S. Nakatsuji, Y.C. Otani, Evaluation of spin diffusion length and spin Hall angle of the antiferromagnetic Weyl semimetal Mn_3Sn. *Phys. Rev. B* **99**, 184425 (2019).

32. J. Liu, L. Balents, Anomalous Hall effect and topological defects in antiferromagnetic weyl semimetals: Mn_3Sn/Ge. *Phys. Rev. Lett.* **119**, 087202 (2017).

33. S. Tomiyoshi, Y. Yamaguchi, Magnetic structure and weak ferromagnetism of Mn_3Sn studied by polarized neutron diffraction. *J. Physical Soc. Japan* **51**, 2478–2486 (1982).

34. N. Sung, F. Ronning, J. Thompson, E. Bauer, Magnetic phase dependence of the anomalous Hall effect in Mn_3Sn single crystals. *Appl. Phys. Lett.* **112**, 132406 (2018).

35. P. J. Brown, V. Nunez, F. Tasset, J. B. Forsyth, P. Radhakrishna, Determination of the magnetic structure of Mn_3Sn using generalized neutron polarization analysis. *J. Phys. Condens. Matter* **2**, 9409–9422 (1990).

36. T. Nagamiya, S. Tomiyoshi, Y. Yamaguchi, Triangular spin configuration and weak ferromagnetism of Mn_3Sn and Mn_3Ge. *Solid State Commun.* **42**, 385–388 (1982).

37. N. Roschewsky, C.-H. Lambert, S. Salahuddin, Spin-orbit torque switching of ultralarge-thickness ferrimagnetic GdFeCo. *Phys. Rev. B* **96**, 064406 (2017).

38. E. Grimaldi, V. Krizakova, G. Sala, F. Yasin, S. Couet, G. Sankar Kar, K. Garello, P. Gambardella, Single-shot dynamics of spin–orbit torque and spin transfer torque switching in three-terminal magnetic tunnel junctions. *Nat. Nanotechnol.* **15**, 111–117 (2020).

39. W. Zhu, L. Seve, R. Sears, B. Sinkovic, S. S. P. Parkin, Field cooling induced changes in the antiferromagnetic structure of nio films. *Phys. Rev. Lett.* **86**, 5389–5392 (2001).

40. J. M. Taylor, E. Lesne, A. Markou, F. K. Dejene, B. Ernst, A. Kalache, K. G. Rana, N. Kumar, P. Werner, C. Felser, S. S. P. Parkin, Epitaxial growth, structural characterization, and exchange bias of noncollinear antiferromagnetic Mn_3Irthin films. *Phys. Rev. Mater.* **3**, 074409 (2019).

41. M. Finazzi, M. Savoini, A. R. Khorsand, A. Tsukamoto, A. Itoh, L. Duò, A. Kirilyuk, T. Rasing, M. Ezawa, Laser-induced magnetic nanostructures with tunable topological properties. *Phys. Rev. Lett.* **110**, 177205 (2013).

42. W. Koshibae, N. Nagaosa, Creation of skyrmions and antiskyrmions by local heating. *Nat. Commun.* **5**, 5148 (2014).

43. S.-G. Je, P. Vallobra, T. Srivastava, J.C. Rojas-Sánchez, T. H. Pham, M. Hehn, G. Malinowski, C. Baraduc, S. Auffret, G. Gaudin, S. Mangin, H. Béa, O. Boulle, Creation of magnetic skyrmion bubble lattices by ultrafast laser in ultrathin films. *Nano Lett.* **18**, 7362–7371 (2018).

44. A. Nordrum, The fight for the future of the disk drive. *IEEE Spectr.* **56**, 44–47 (2019).

45. C. Hahn, G. Wolf, B. Kardasz, S. Watts, M. Pinarbasi, A. D. Kent, Time-resolved studies of the spin-transfer reversal mechanism in perpendicularly magnetized magnetic tunnel junctions. *Phys. Rev. B* **94**, 214432 (2016).

46. H. Tomita, K. Konishi, T. Nozaki, H. Kubota, A. Fukushima, K. Yakushiji, S. Yuasa, Y. Nakatani, T. Shinjo, M. Shiraishi, Y. Suzuki, Single-shot measurements of spin-transfer switching in CoFeB/MgO/CoFeB magnetic tunnel junctions. *Appl. Phys. Express* **1**, 061303 (2008).

47. T. Higo, D. Qu, Y. Li, C. L. Chien, Y. Otani, S. Nakatsuji, Anomalous Hall effect in thin films of the Weyl antiferromagnet Mn_3Sn. *Appl. Phys. Lett.* **113**, 202402 (2018).

48. J. C. Slonczewski, Current-driven excitation of magnetic multilayers. *J. Magn. Magn. Mater.* **159**, L1–L7 (1996).

49. W. H. Zachariasen, *Theory of X-Ray Diffraction in Crystals* (Dover Publications Inc., 1994).

50. J. M. Taylor, *A. Markou, E. Lesne, P. K. Sivakumar, C. Luo, F. Radu, P. Werner, C. Felser, S. S. P. Parkin*, Anomalous and topological Hall effects in epitaxial thin films of the noncollinear antiferromagnet Mn_3Sn. *Phys. Rev. B* **101**, 094404 (2020).

51. Y. You, X. Chen, X. Zhou, Y. Gu, R. Zhang, F. Pan, C. Song, Anomalous Hall effect–like behavior with in-plane magnetic field in noncollinear antiferromagnetic Mn_3Sn films. *Adv. Electron. Mater.* **5**, 1800818 (2019).
